# Supplementary material for: Multi-faceted epigenetic dysregulation of gene expression promotes esophageal squamous cell carcinoma
Source: Nat Commun. 2020 Jul 22;11:3675. doi: 10.1038/s41467-020-17227-z (PMC7376194; doi:10.1038/s41467-020-17227-z)
Supplement: Supplementary file 1 — Supplementary Information [file 41467_2020_17227_MOESM1_ESM.pdf]

Multi-faceted epigenetic dysregulation of gene expression promotes esophageal squamous cell carcinoma

Cao, Lee, Wu, Zaman (co-first authors) et al,

a

	Sex	Age	Smoking	Drink (Y/N)	Family history (Y/N)	Diagnosis	Pathological stage	TNM stage	Lymph node metastasis (Y/N)	Survival time (months)
**Patient 1**	Male	61	20 cigarettes/ day, 40 years	N	N	ESCC	Moderately differentiated	T3N1aM0	Y	Alive
**Patient 2**	Female	77	N	N	Y	ESCC	Highly or moderately differentiated	T3N0M0	N	Alive
**Patient 3**	Male	56	20 cigarettes/ day, 30 years	N	Y	ESCC	Moderately differentiated	T2N0M0	N	6
**Patient 4**	Female	58	N	N	N	ESCC	Moderately differentiated	T3N1aM0	Y	21
**Patient 5**	Male	59	20 cigarettes/ day, 30 years	N	N	ESCC	Moderately differentiated	T3N0M0	N	Alive
**Patient 6**	Male	63	N	N	Y	ESCC	Moderately or poorly differentiated	T3N0M0	N	Alive
**Patient 7**	Male	60	15 cigarettes/ day, 25 years	N	N	ESCC	Moderately differentiated	T3N0M0	N	Alive
**Patient 8**	Male	65	N	N	Y	ESCC	Highly or moderately differentiated	T2N1aM0	Y	23
**Patient 9**	Male	70	20 cigarettes/ day, 40 years	N	N	ESCC	Highly differentiated	T2N0M0	Y	Alive
**Patient 10**	Female	58	N	N	N	ESCC	Moderately differentiated	T1bN0M0	Y	Alive

b

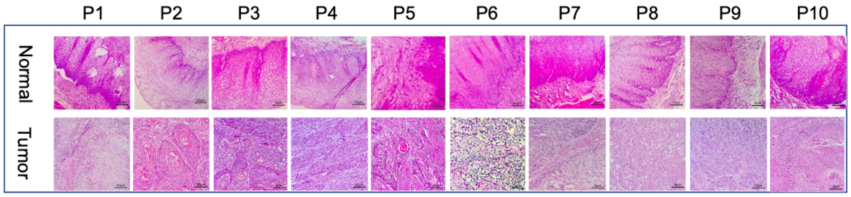

Supplementary Figure 1. Clinical information and histological images. (a) The clinical information from ten patients with esophageal squamous cell carcinoma (ESCC). Patients provided consent to publish de-identified information in the study. (b) Histological slides with hematoxylin and eosin (H & E) staining derived from ten surgically resected ESCC tumor specimens and adjacent normal tissues. Two independent pathologists evaluated the diagnoses. Scale bar: 50 µm.

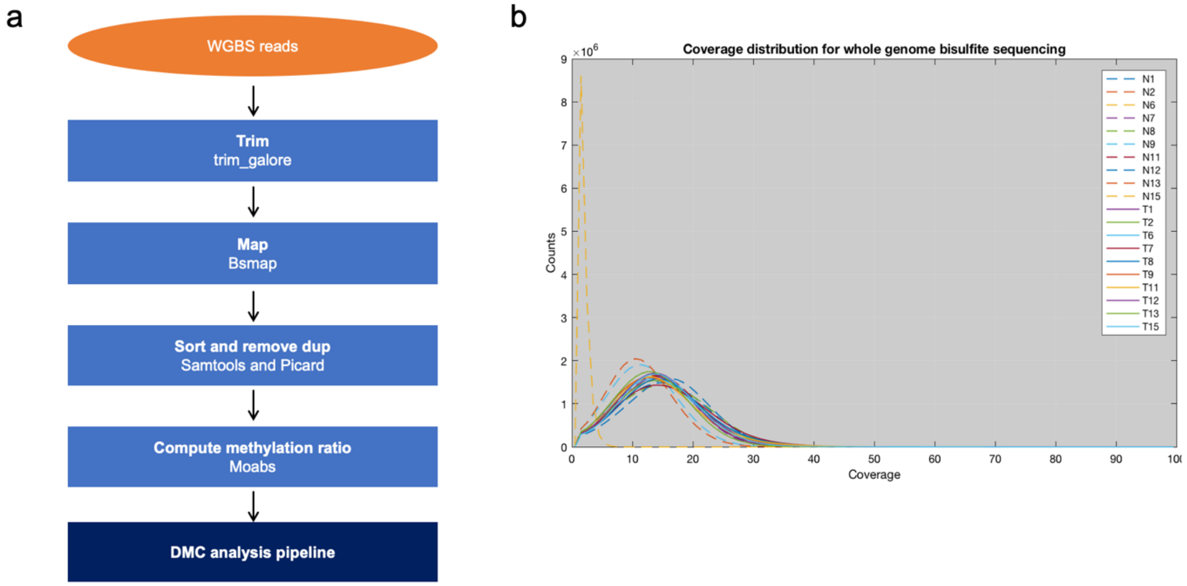

Supplementary Figure 2. Pipeline of whole genome bisulfite sequencing (WGBS) analysis. (a) The pipeline of WGBS analysis is shown. (b) The read coverage distribution of twenty WGBS samples is shown. One normal sample was filtered out due to low read coverage. Ten tumor samples and nine normal samples were used for downstream analyses.

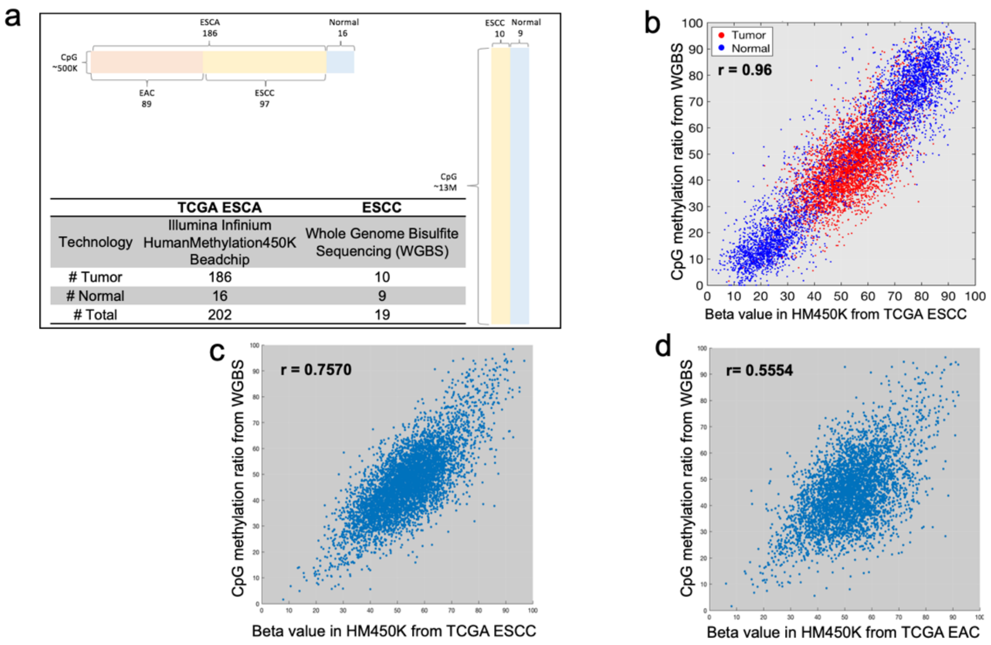

Supplementary Figure 3. Correlation analysis between our WGBS data and TCGA-ESCA methylation data. (a) Our WGBS data consists of 19 samples (9 normal and 10 tumor samples). TCGA-ESCA HM450K dataset is comprised of 16 normal samples, 186 tumor samples; among tumor samples, 89 are esophageal adenocarcinoma (EAD), 97 are esophageal squamous cell carcinoma (ESCC). (b) The CpG methylation status detected by our ESCC WGBS dataset and TCGA-ESCA HM450K dataset (N=202) is highly correlated (Pearson correlation r=0.96). Red dots represent CpG methylation status in tumors, blue dots represent CpG methylation status in normal samples. (c) The correlation of CpG methylation status detected by our ESCC WGBS (n=10) and TCGA-ESCC HM450K samples (n=97) is shown. Pearson correlation efficient = 0.757. (d) The correlation of CpG methylation status detected by our ESCC WGBS (n=10) and TCGA-EAC HM450K samples (n=89). Pearson correlation efficient = 0.554.

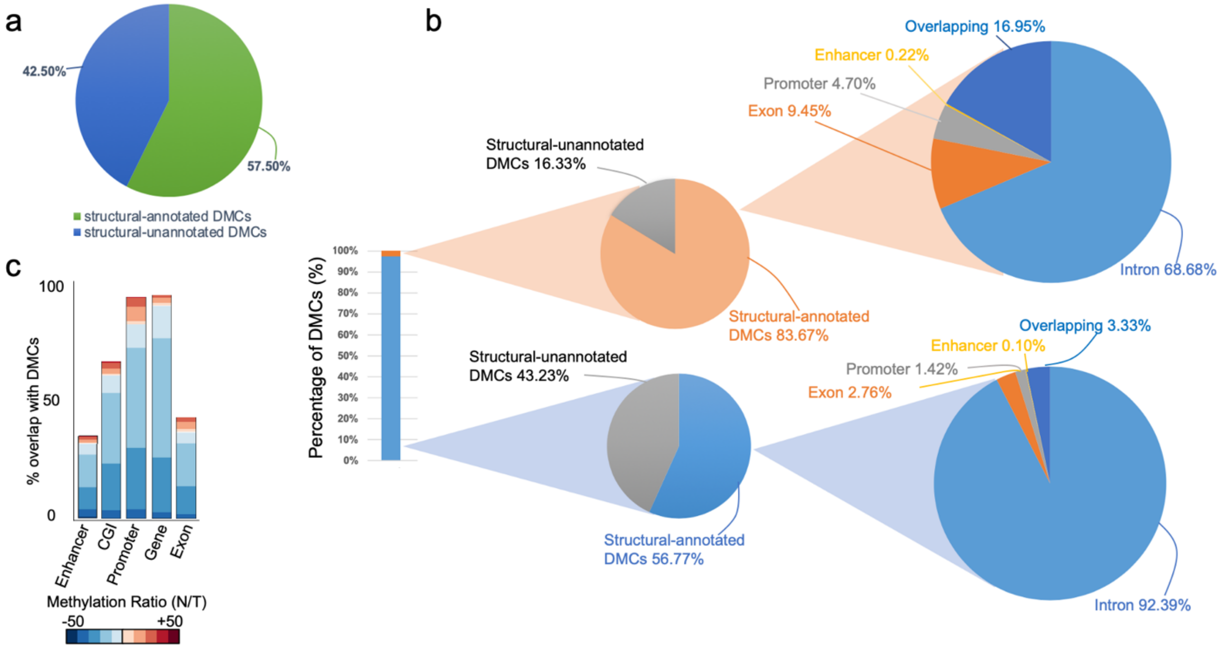

Supplementary Figure 4. Characterization of differentially methylated CpGs (DMCs) in ESCC. (a) 57.50% out of 5,092,845 DMCs are mapped to annotated regions defined in the ENCODE project. (b) Among these DMCs, 97.3% are hypomethylated CpGs (blue color in bar plot) and only 2.7% are hypermethylated CpGs (red color in the bar plot). 83.67% of hypermethylated DMCs are overlapping with well-annotated regions such as introns, exons, promoters and enhancer regions (pie chart on top panel). 55.77% of hypomethylated DMCs are overlapping with well-annotated regions (pie chart on lower panel). (c) Regulatory elements overlapped with DMCs are identified. 99% of genes overlap with DMCs; 98% of promoters overlap with DMCs; 70% of CGI has at least one on DMCs; 43% of exons overlap with DMCs, and 36% of enhancers overlap with DMCs. Overlap with hypomethylated DMCs are blue, and overlaps with hypermethylated DMCs are red.

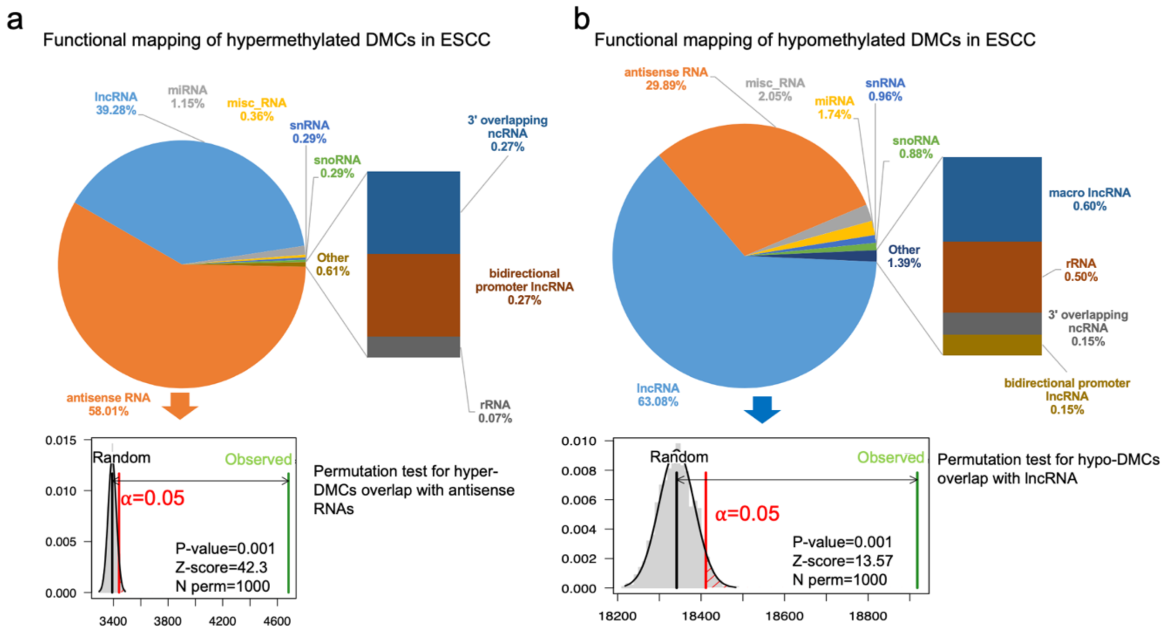

Supplementary Figure 5. Functional annotation of differentially methylated CpGs (DMCs). (a) 58% of hypermethylated DMCs (hyper-DMCs) are overlapping with regions encoding antisense RNA (orange color), while 39% map to regions containing long non-coding RNAs (lncRNA) (blue color), and the remaining DMCs are located in regions harboring other non-coding RNAs such as microRNAs (miRNAs) and small nuclear RNAs (snRNAs). (b) 63.08% of hypomethylated DMCs (hypo-DMCs) are overlapping with regions encoding lncRNA (blue color), while 29.89% map to regions containing antisense RNAs (orange color). The remaining DMCs are located in regions harboring other non-coding RNAs. The permutation test (regioneR package) was performed for the statistical difference between hyper-DMCs and antisense RNA overlapping (p=0.001, Z-score=42.3, 1000 permutation) or hypo-DMCs and lncRNA overlapping (p=0.001, Z-score=13.57, 1000 permutation).

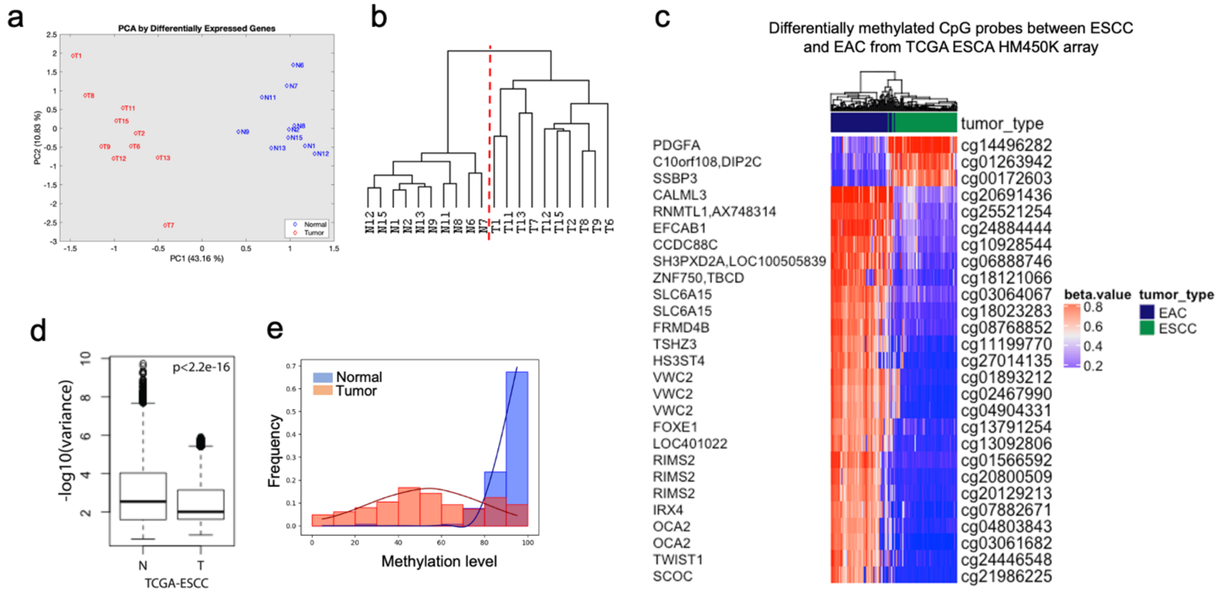

Supplementary Figure 6. Variance of DNA methylation and gene expression in ESCC. (a) Principal Component Analysis (PCA) for RNA-seq derived from ten matched normal-ESCC tumor samples. Normal and ESCC tissues have distinct transcriptomes, which separate them into two distinct cell states. Blue color represents normal samples, red color represents tumor samples. (b) Dendrogram derived from Hierarchical Cluster Analysis (HCA) with Euclidean distance on RNA-seq data showed distinct normal and ESCC groups. (c) Hierarchical clustering of differentially methylated CpGs (DMCs) in ESCC relative to EAC. TCGA-ESCA HM450K methylation data includes 97 ESCC and 89 EAC samples. There is a total of 381,132 CpGs probes with normalized β value in each sample after data processing. A linear model limma was used to define DMCs (n=27) with the criteria of |log2(FC)| >= 0.59 and FDR < 0.05. The CpG probes are listed on the right side and their associated genes are listed on the left side of heatmap. Samples in the column are clustered as either ESCC (green bar) or EAC (blue bar). (d) The higher variance of CpG methylation in TCGA-ESCC samples (n=97) relative to normal samples (n=5) is shown. The CpG probes from HM450K methylation arrays for TCGA-ESCC samples were computed as the means of variance and standard deviation and -log10(variance) was used for plotting. variance with the formula $s\hat{}2 = 1/(n-1)\sum_{i=1}^{n}(x_{i}-\bar{x})2$. Box and whisker plot: center line, median; box limits, upper and lower quartiles; and whiskers, maximum and minimum values, circle dots, outliers. Statistical significance was assessed by two-sided t-test, p<2.2e-16. (e) Simulations of Ornstein-Uhlenbeck processes was performed for CpG islands at selected promoter regions in both normal (n=9) and cancer samples (n=10). The histograms of the levels of the of the hypermethylated ESCCAL-1 (CASC9) promoter (Chr8: 76,190,572-76,190,784) are overlaid with the density plots of the simulations. Red bars indicate CpG methylation status in tumor samples, and blue bars indicate CpG methylation status in normal samples.

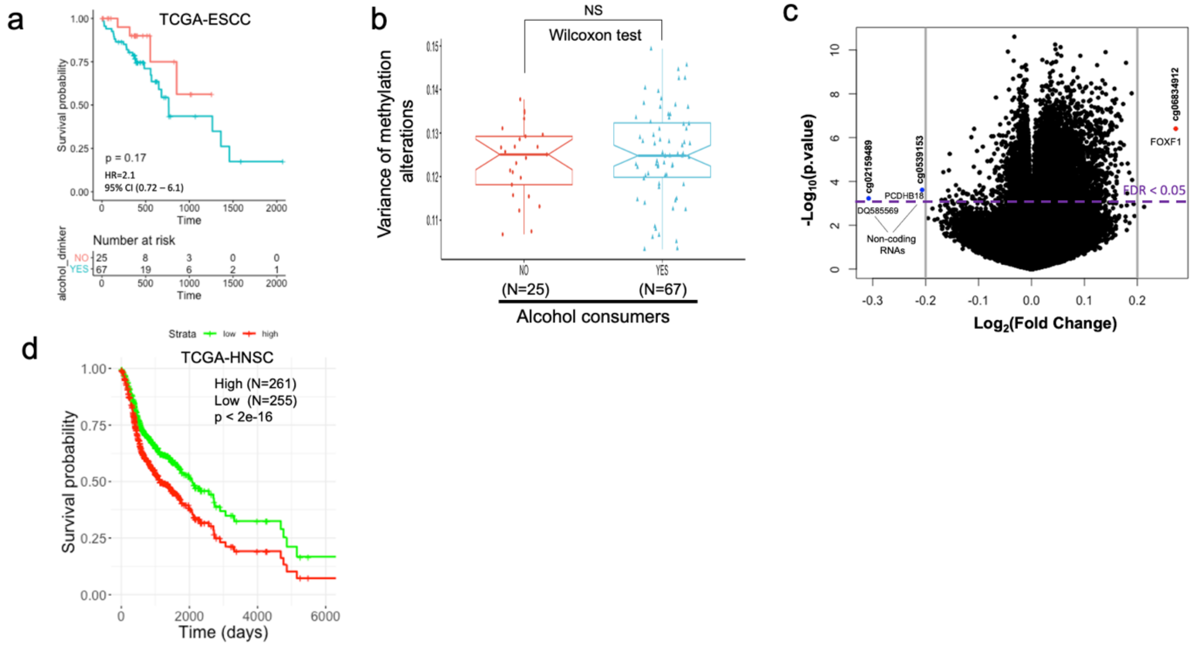

Supplementary Figure 7. The impact of alcohol-intake on survival, DNA methylation and variance of DNA methylation. (a) In the TCGA ESCC cohort (n=97), 70 patients have an alcohol consumption history, 25 are without an alcohol consumption history, 2 samples are unannotated for alcohol consumption. Filtering out 3 additional cases lacking follow-up data resulted in 92 samples that could be used for analysis. The Kaplan-Meier survival curve showed a trend toward inferior overall survival time in patients with alcohol consumers compared to non-alcohol consumers, log-rank test, p-value = 0.17, HR=2.01, 95% CI [0.72-6.1]. (b) Boxplot shows the variance of all DNA methylation changes in ESCC patients with either alcohol consumers (N=67) or non-alcohol consumers (N=25) in TCGA-ESCC cohort. Statistical significance was assessed by two-sided Wilcoxon rank-sum test. p = 0.89; NS: no significance. (c) Volcano plot of differentially methylated probes between alcohol-user group and non-alcohol-user group in TCGA-ESCC HM450K dataset. Each dot represents individual probes in HM450K array. Criteria for significantly differentially methylated probes is |log2(fold-change)| > 0.2 AND adjusted p.value < 0.05. The red dot = significantly hypermethylated probe; the blue dots = significantly hypomethylated probes. (d) Multi-variate Kaplan Meier plot shows higher variance of DNA methylation contributes to poor survival in TCGA-Head and Neck squamous carcinoma (N=516). Median of variance separates the two groups. Log-rank test, p < 2e-16.

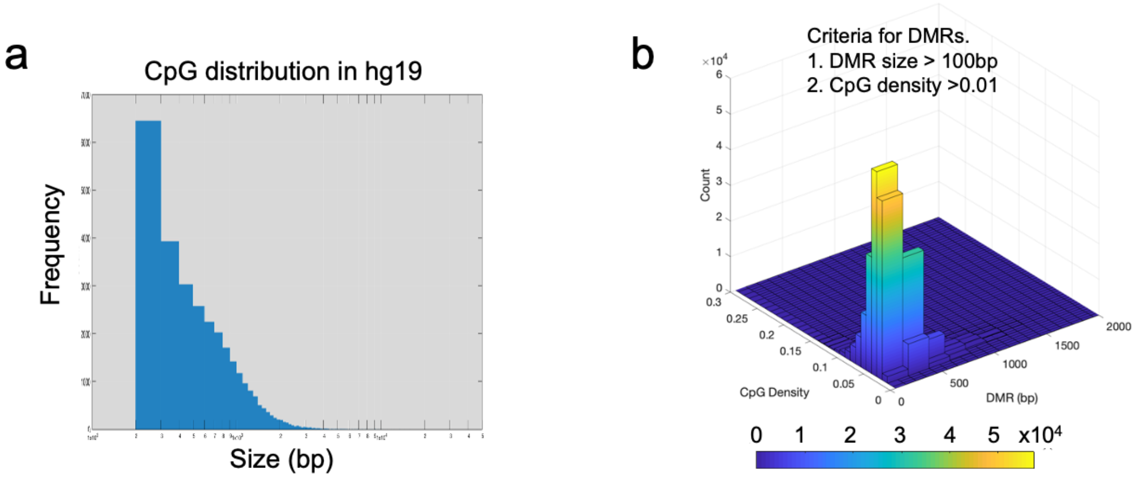

Supplementary Figure 8. **Identification and characterization of differentially methylated regions (DMRs).** (a) CpG distribution in human reference genome (hg19) is shown. (b) Bivariate 3D histogram shows the distribution of DMRs in terms of DMR base-pair size and CpG density. Two criteria were used for defining DMRs: the length of DMRs is greater than 150 base pairs and CpG density within the region is greater than 1%.

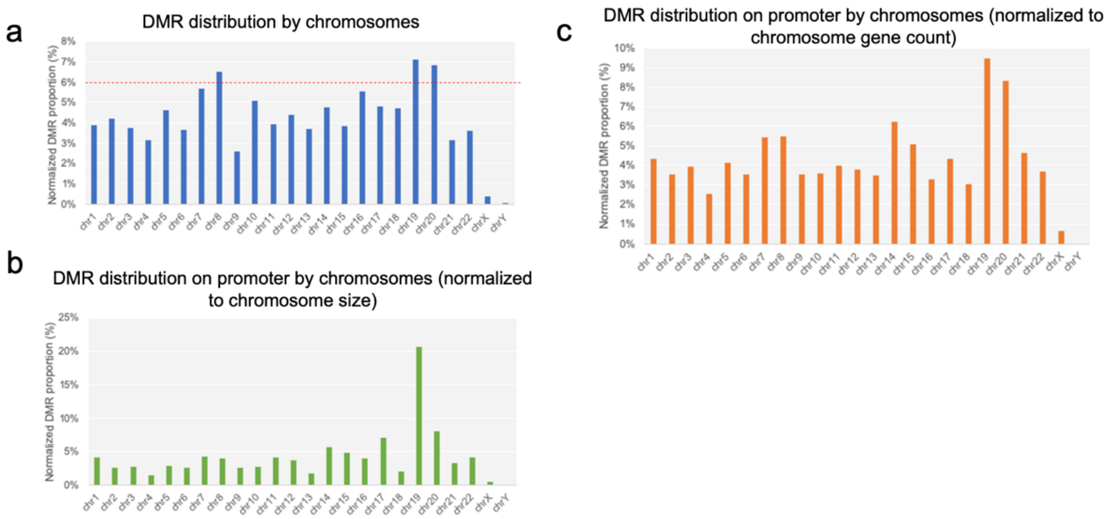

Supplementary Figure 9. The distribution of Differentially Methylated Regions (DMRs) in chromosomes and gene promoters. (a) Normalized distribution of DMRs across chromosomes. (b) Distributions of DMRs in promoter regions across chromosomes normalized by chromosomal size. (c) Distributions of DMRs in promoter regions across chromosomes normalized by gene numbers in each chromosomes.

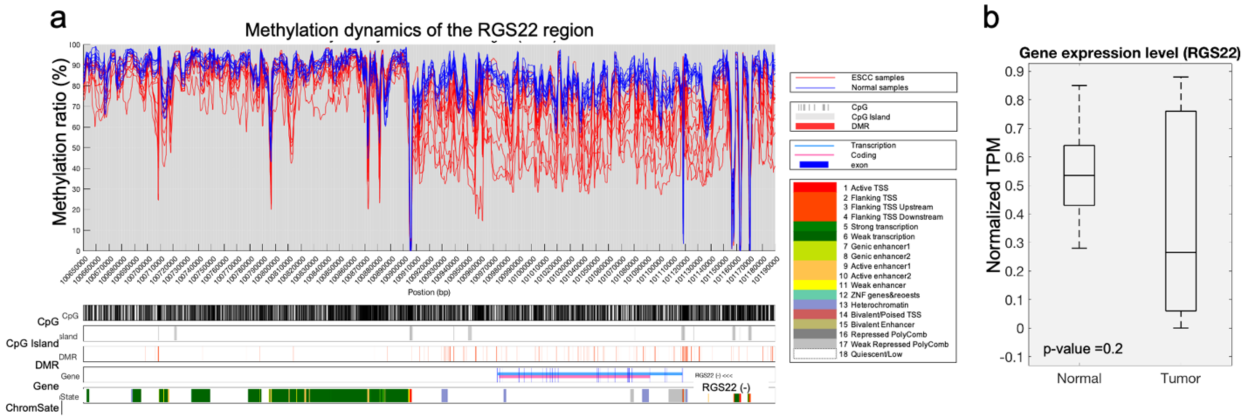

Supplementary Figure 10. The representative genomic region with hypomethylation across the gene body. (a) RGS22 (regulator of G protein signaling) shows a notable gene body methylation difference between ESCC (red lines) and normal esophageal tissues (blue lines) (top panel). Five different tacks (CpGs, CpG island, DMRs, gene transcript and ChromState show their locations across the defined genomic regions. (b) Gene expression of RGS22 in ESCC tumor (n=10) and normal samples (n=10) from RNA-seq data. Box and whisker plot: center line, median; box limits, upper and lower quartiles; and whiskers, maximum and minimum values. Statistical significance was assessed by two-sided t-test, p-value = 0.2.

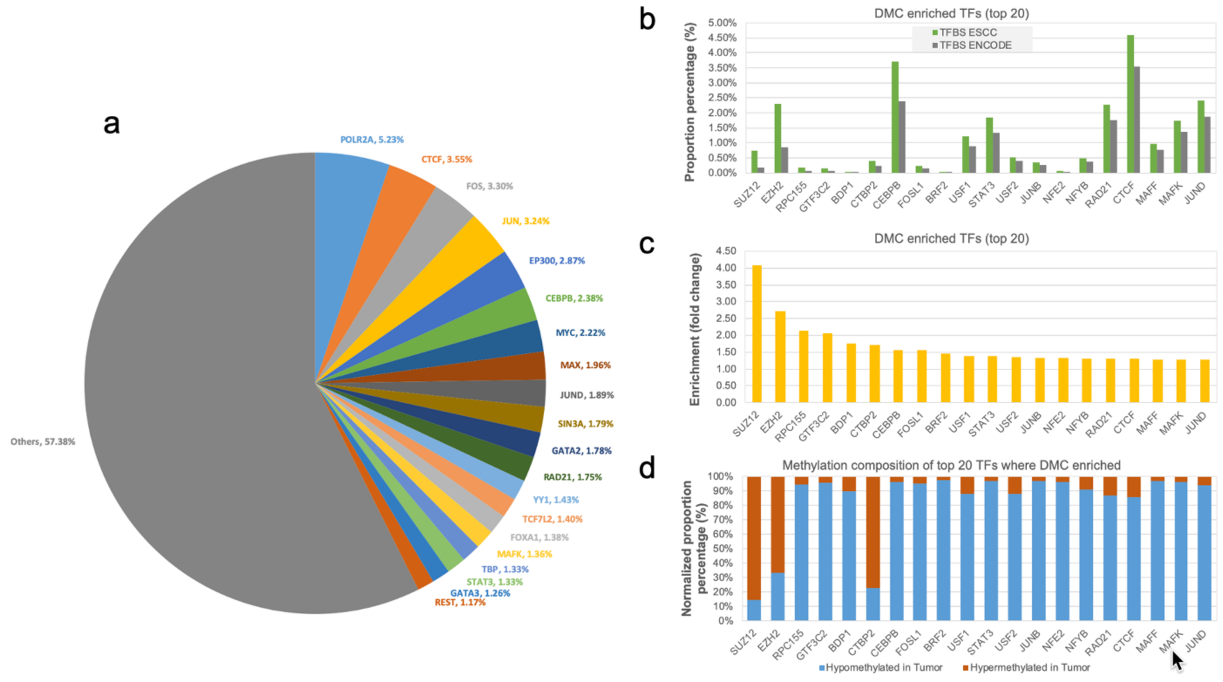

Supplementary Figure 11. **Transcription factors (TFs) from the ENCODE project and CpG content in their binding consensus.** (a) The frequency of each TF binding sited in the genome is shown. 161 TFs from the ENCODE project were used to compute the level of binding consensus across the genome. (b) The top 20 TFs whose binding consensus are affected by changings in CpG methylation in ESCC (green bars) versus that in reference hg19 genome (grey bars). (c) The ratio of the top 20 enriched TF between ESCC and reference genome. SUZ12 is the most enriched TF, followed by EZH2. Both are components of the PRC2 (Polycomb Repressive Complex 2). (d) DMC distribution in top 20 TFs binding consensus sites; red portion shows the hypermethylated DMCs and the blue portion shows hypomethylated DMCs. EZH2, SUZ12 and CTBP2 binding consensus sequences harbor more than 60% of hypermethylated CpGs.

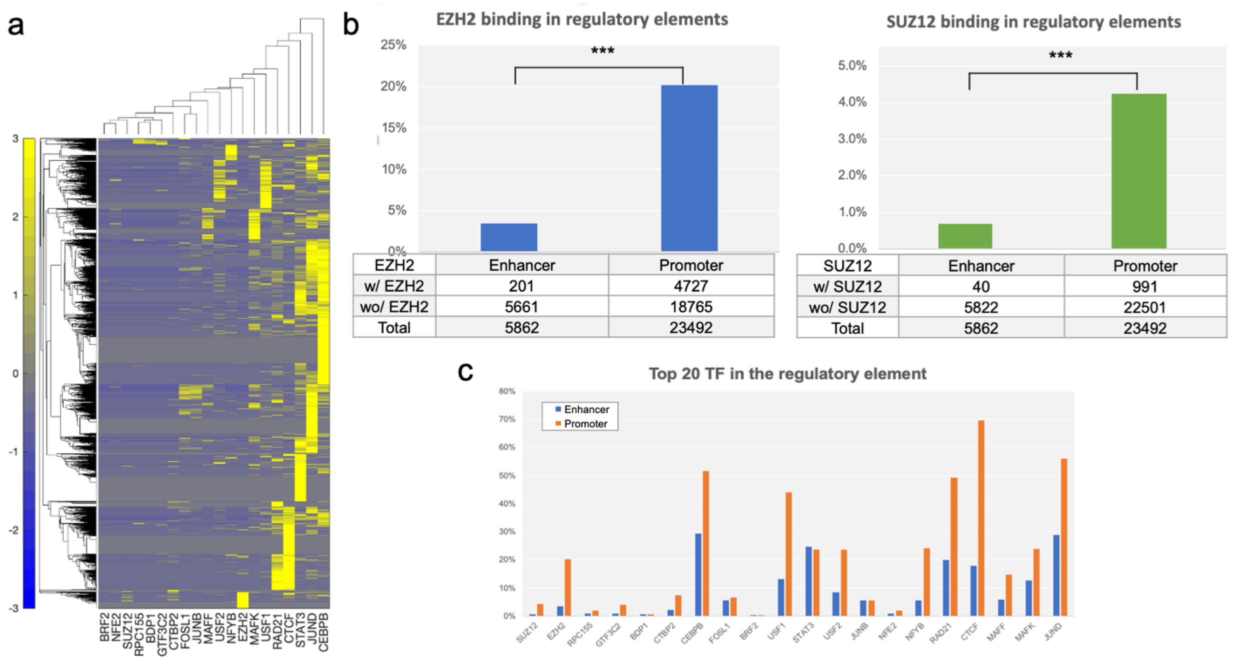

Supplementary Figure 12. Comparison of EZH2, SUZ12 and other TFs binding preferences in enhancers and promoters. (a) Top 20 transcription factors were associated with enhancers given binding scores retrieved from ENCODE Project. (b) The binding preference of EZH2 and SUZ12 on promoter s and enhancers. Among 23.5K genes, 20% of genes have EZH2 binding sites in their promoters while 3% of enhancers have EZH2 binding sites. 4% of genes have SUZ12 binding sites in their promoters while 0.7% of enhancers have SUZ12 binding sites. One-way ANOVA, *** p-value < 0.001. (c) Enrichment of top 20 TFs in both promoters and enhancers are studied. Except for STAT3 and JUNB, most of top 20 TFs enriched in the promotor regions.

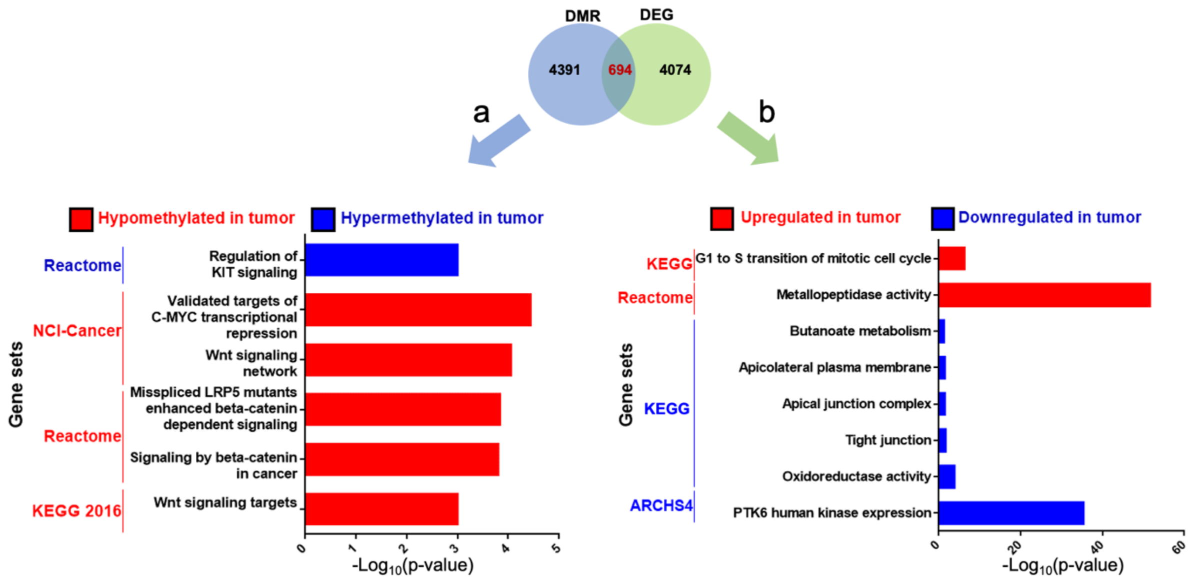

Supplementary Figure 13. Functional annotation of gene set defines biological pathways. (a) 4391 genes whose promoter regions display differentially methylation changes between ESCC tumors and adjacent normal samples from whole genome bisulfite sequencing were identified. (b) 4768 differentially expressed genes were derived from RNAseq analysis of ESCC tumors and adjacent normal samples. The Functional annotation was carried out using a hypergeometric test in multiple databases (e.g. GO molecular function, GO biological process, WikiPathways, Reactome, KEGG 2016, BIOGRID, ARCHS4, NCI-Cancer). The data are represented as a bar diagram indicating the probability of the gene set enrichment metric $-\text{Log}_{10}$ (p-value). An adjusted p value cutoff for significant gene sets was set to 0.01 ($-\text{Log}_{10}$ (p-value)>2). Hypergeometric test was used for pathway enrichment analysis within the algorithm.

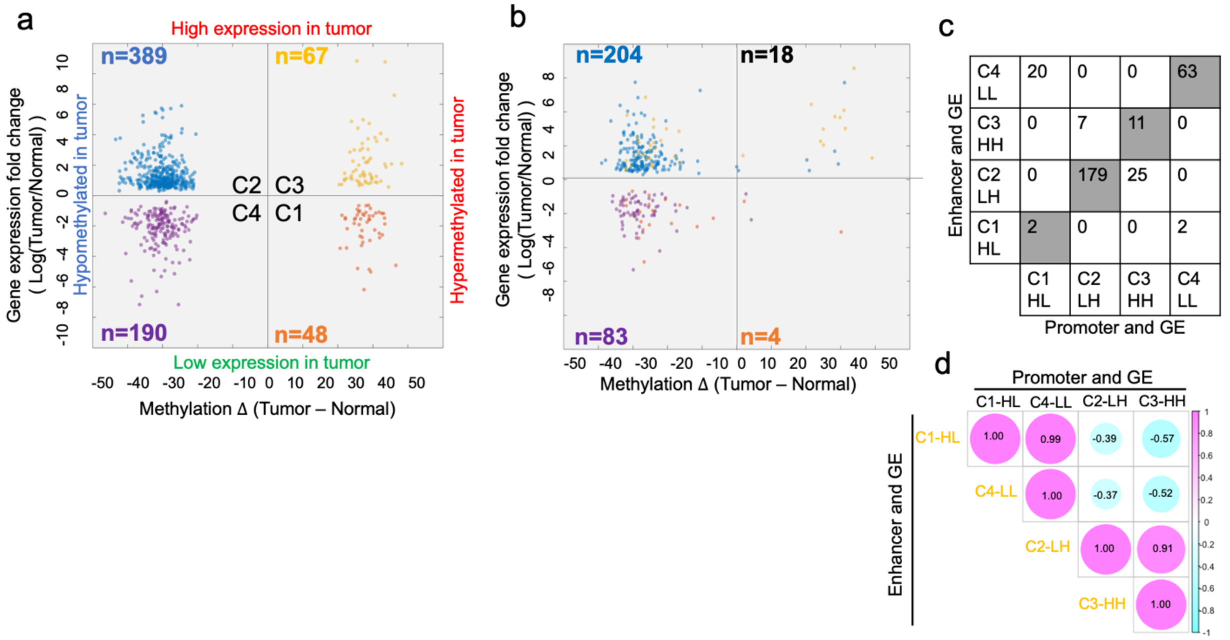

Supplementary Figure 14. The patterns of differentially methylated promoter- or enhancer-associated gene expressions and their correlations. (a) 694 genes whose promoter regions (-4500 to + 500 base pairs relative to transcription start site (TSS)) are significantly differentially methylated and their expression levels are differentially changed in ESCC relative to normal samples. The genes were systematically classified into four distinct clusters (denoted as C1, C2, C3 and C4) according to the methylation (met) and gene expression (ge) pattern. C1(n=48, $H_{met}L_{ge}$) showed hypermethylated promoters with decreased ge; C2(n=389, $L_{met}H_{ge}$) showed hypomethylated promoters with increased ge; C3(n=67, $H_{met}H_{ge}$) contained hypermethylated promoters with increased ge; C4(n=190, $L_{met}L_{ge}$) denoted hypomethylated promoters with decreased ge. (b) Enhancer regions from the latest annotation of **HACER**, an atlas of **H**uman **AC**tive **E**nhancer to interpret **R**egulatory variants (http://bioinfo.vanderbilt.edu/AE/HACER/) were obtained. 534 out of 694 genes have confirmed distal regulatory enhancers. Methylation delta in enhancer and gene expression fold changed of the closest gene are associated. Total 309 genes have enhancer annotation. Identified C1-C4 genes were mapped into enhancer and gene expression space. (c) Membership based on promoter methylation and gene expression dynamically rearranged based on enhancer methylation and gene expression. Most of them stayed in the same planes but most of C1 plane moved to C4 plane and most of C3 planes moved to C2 plane. (d) Pearson correlation analysis shows high correlation between enhancer-gene expression pattern and promoter-gene expression pattern (see diagonal circles) with the exception in non-canonical C3 and C4.

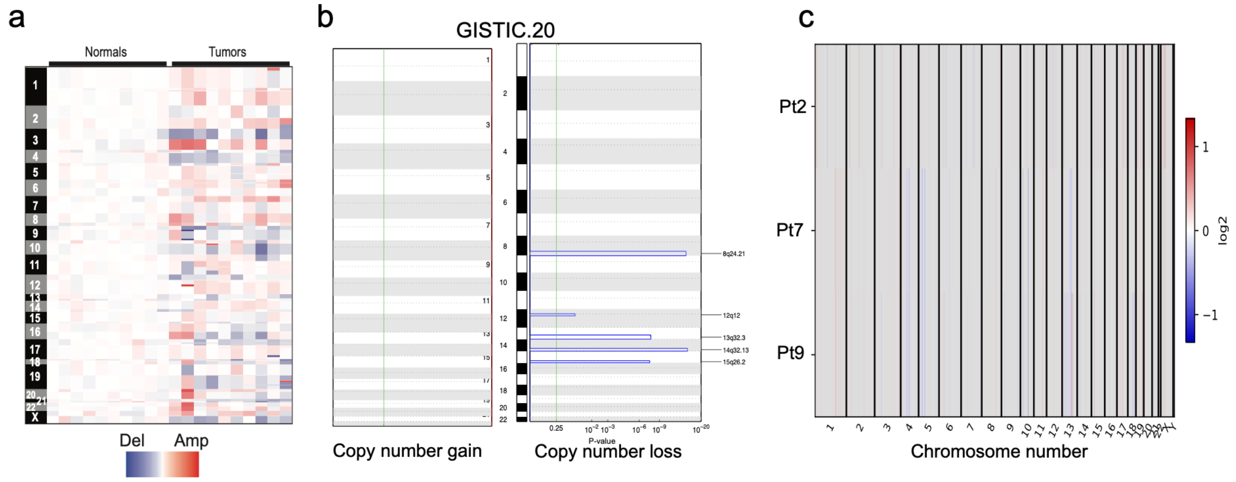

Supplementary Figure 15. Copy number alterations (CNAs) inferred from RNAseq data and WGS. (a) The heatmap of CNA between normal (n=10) and ESCC samples (n=10). Red: copy number gain, blue: copy number loss. (b) Recurrent significant CNAs defined by GISTIC 2.0 algorithm. (c) The copy number variations (CNVs) were analyzed from WGS data derived from three ESCC patient samples. The segments were plotted using "cnvkit" algorithms. Red color indicates copy number gain, blue color indicates copy number loss.

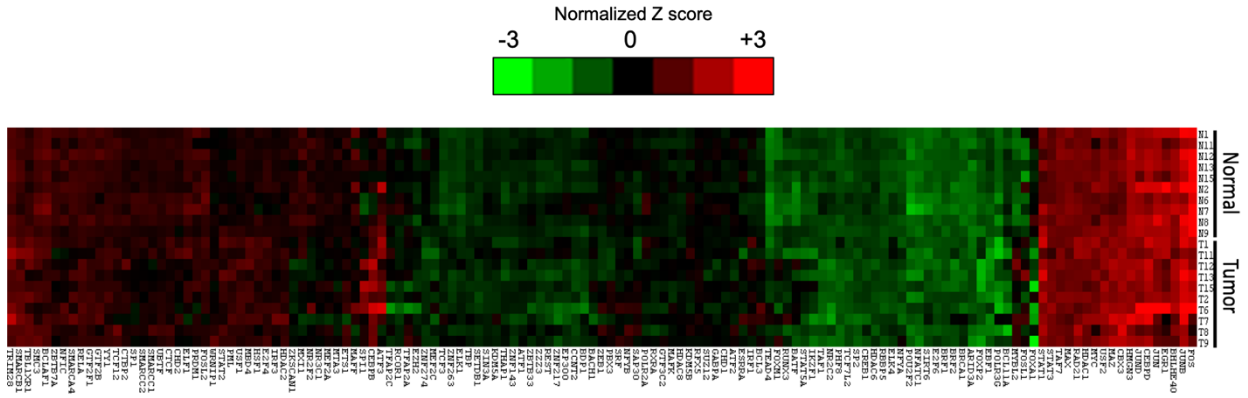

Supplementary Figure 16. Expression of transcription factors across the normal and ESCC tumor samples. The expression of transcription factors (n=161, ENCODE) in esophageal squamous cell carcinoma and adjacent normal tissues. The normalized TPM of transcripts from RNA-seq were used to generate the heatmap. Red color- high expression; green color-low expression.

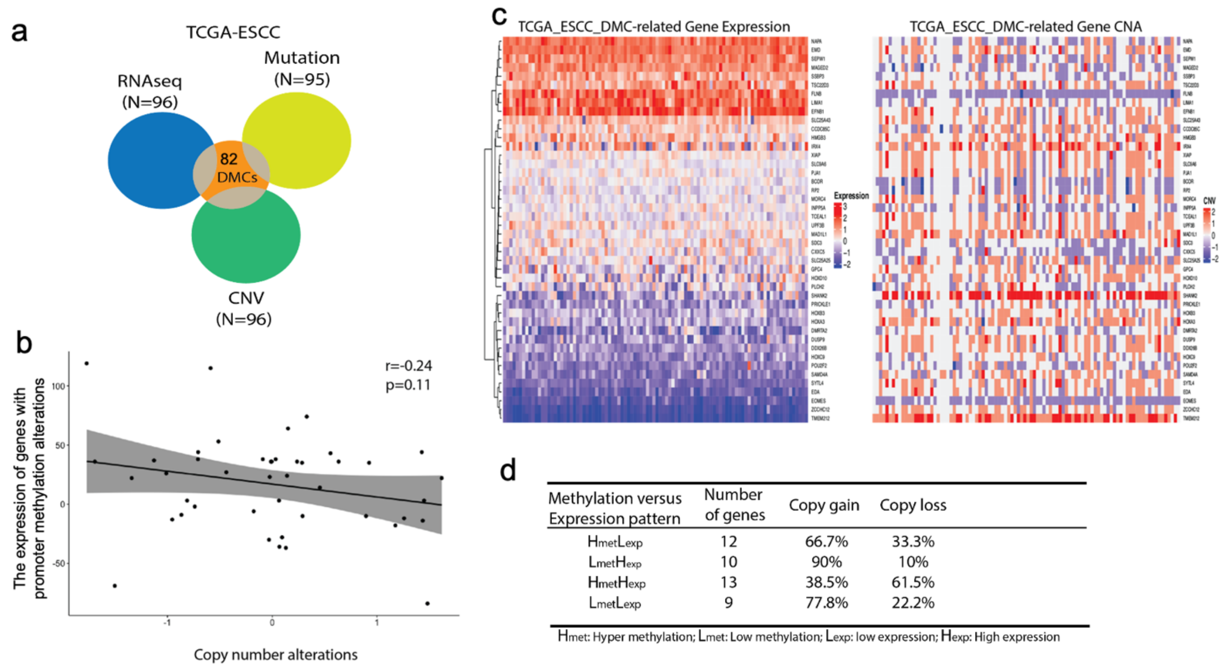

Supplementary Figure 17. Integrative analysis of TCGA-ESCC data for methylation-gene expression clustering. (a) Venn diagram indicates intersection of multi-omics in TCGA-ESCC dataset. (b) The correlation between copy number alteration (CNA) and corresponding gene expression is shown. Spearman coefficient = 0.24, p-value = 0.11, grep error band indicated 95% CI. (c) Heatmap of differential methylated CpGs in promoters and their CNAs is shown. (d) List of gene expression and CNAs in each cluster.

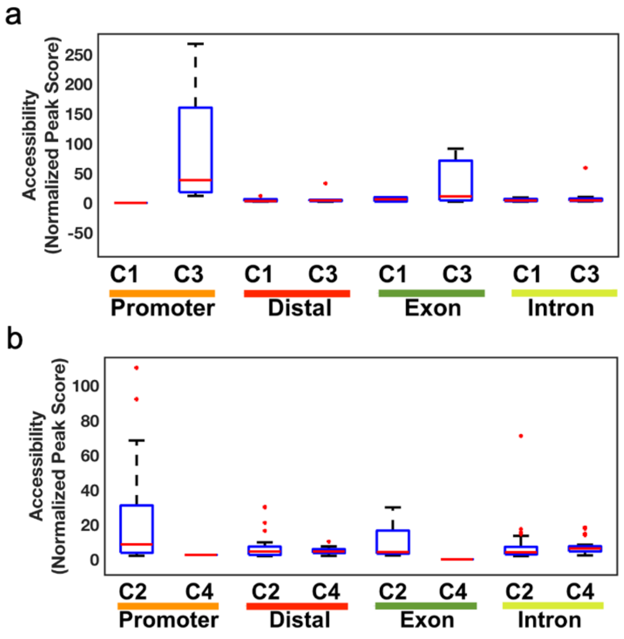

Supplementary Figure 18. Integration of TCGA-ESCA accessibility and four promoter methylation-mediated gene expression clusters in our study. 13,000 TCGA-ESCA chromatin accessible regions were determined by ATAC-seq (Corces MR *et al.* Science. 2018. PMID:30361341). (a) The overlap the chromatin accessible regions with 694 genes is shown. Accessibility is higher in gene promoter and exons in C3 (hypermethylated promoters with increased gene expression) compared to C1 (hypermethylated promoters with decreased gene expression). (b) Accessibility is higher in gene promoter and exons in C2 (hypomethylated promoters with increased gene expression) compared to C4 (hypomethylated promoters with decreased gene expression). Box and whisker plot: center line, median; box limits, upper and lower quartiles; and whiskers, maximum and minimum values, red dots, outliers.

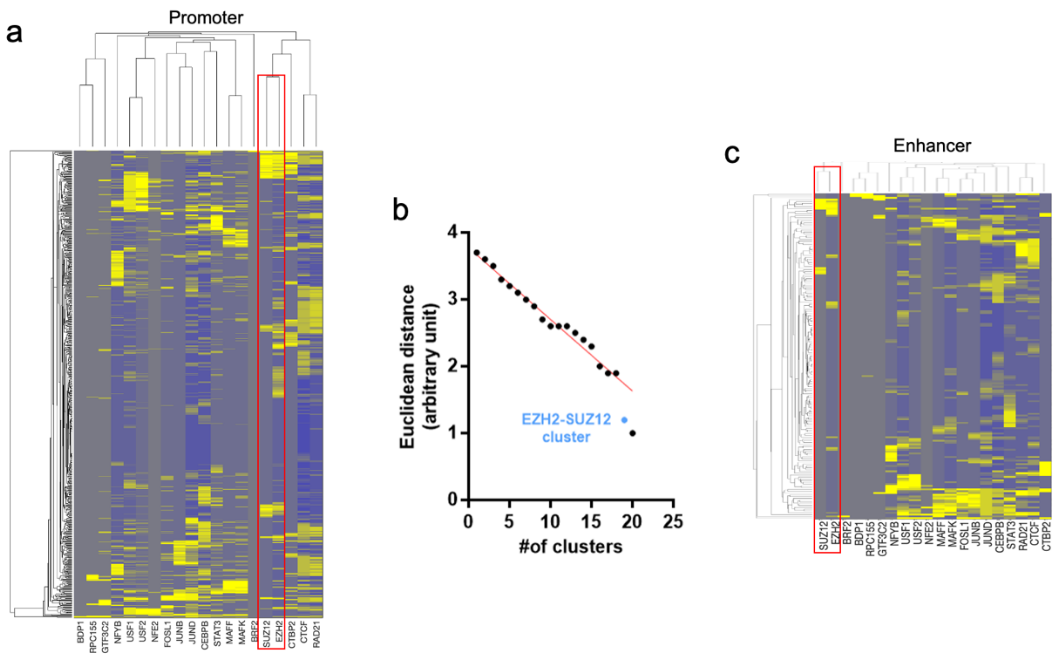

Supplementary Figure 19. Transcription factor binding profiles in promoters and enhancers.
(a) Binding scores of the top 20 TFs in promoter regions are associated with the 694 genes and hierarchically clustered by column normalization. EZH2 and SUZ12 clustered together as components of PRC2 (Polycomb Repressive Complex 2). (b) Dendrogram in main Figure 4d was used for each counts of cluster per Euclidean distance unit (arbitrary distance to scale). An inverse regression line (red) indicates the internal noise of the dendrogram. EZH2-SUZ12 cluster was an outlier from the distribution by measuring interquartile range analysis. (c) Binding scores of the top 20 TFs in enhancer regions are associated with enhancer-annotated 309 genes and are hierarchically clustered by column normalization. EZH2 and SUZ12 clustered together as components of PRC2.

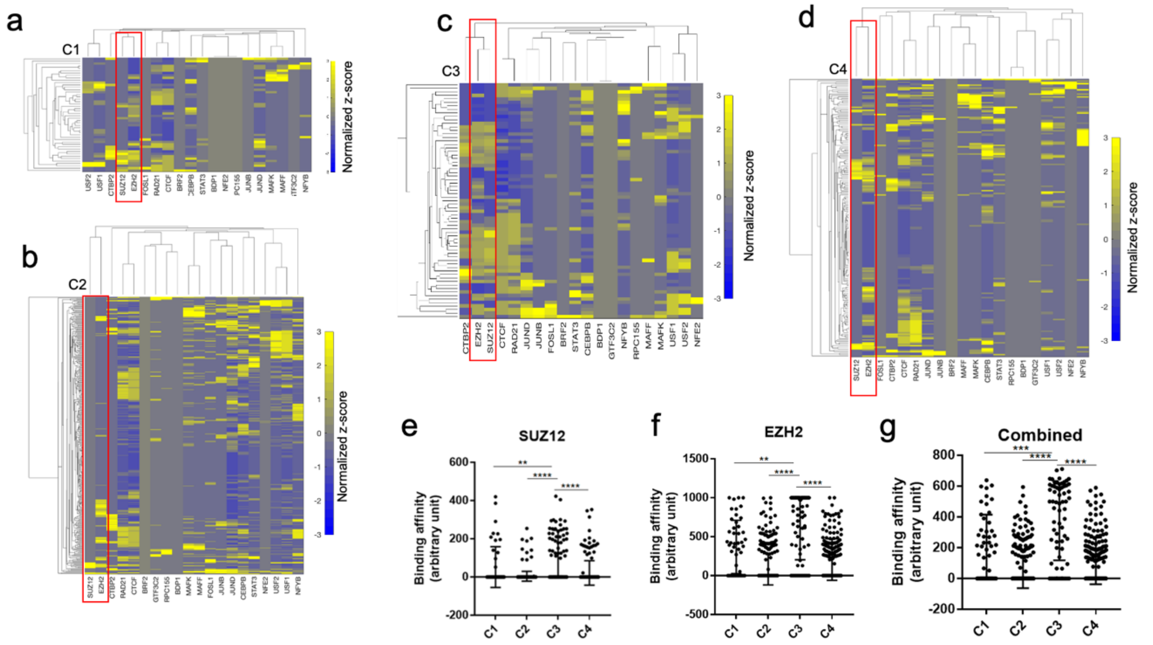

Supplementary Figure 20. Selected transcription factor binding affinities in C1-C4 gene promoters. (a-d) Unsupervised hierarchical clustering for column normalized binding affinity of transcription factors on C1, C2, C3 and C4 gene promoters. The average Euclidean distance was used for the analysis. Data were normalized along the columns where blue represents weak binding between a gene and a transcription factor while yellow represents a strong binding based on z-score showing in the color bar (Dunham I. et al. Nature. 2012. PMID:22955616). (e-g) Quantification of SUZ12, EZH12 or combined affinities in C3 (n=67) compared to other clusters [C1(n=48), C2(n=389) and C4(n=190)]. Error bars are median values with 95% confidence intervals. Statistical significance was measured by unpaired two-sided t-test, ** p-value < 0.01, *** p-value < 0.005, **** p-value < 0.001.

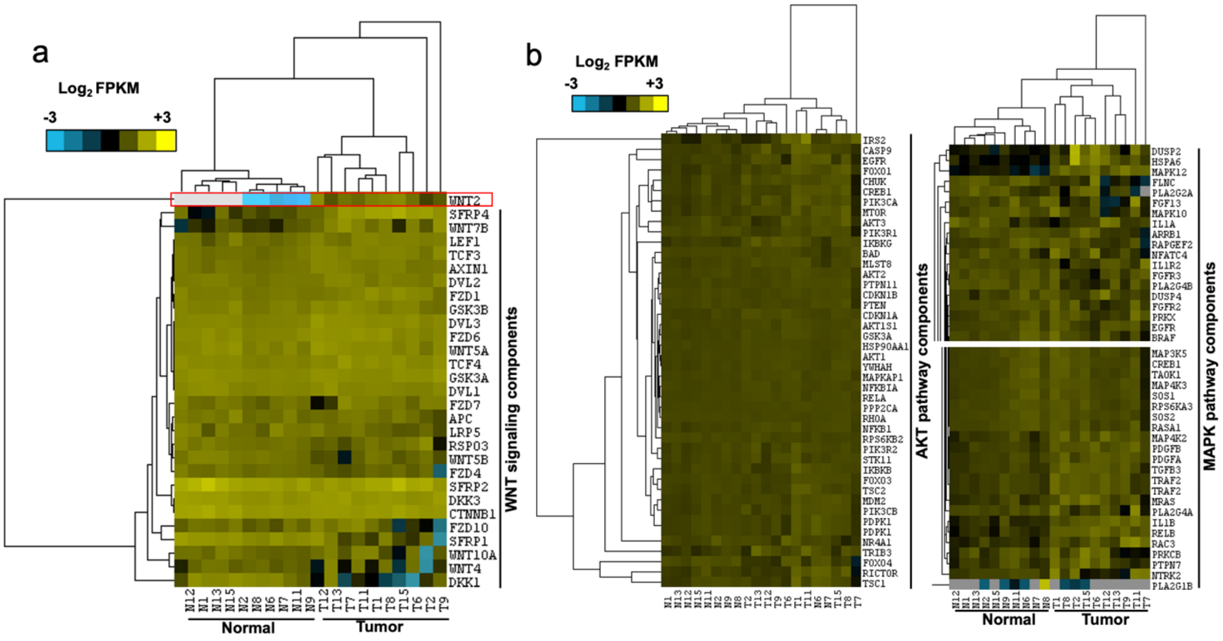

Supplementary Figure 21. Gene expression in oncogenic pathways derived from RNAseq data in matched normal and ESCC tumor samples. (a) Heatmap shows specific upregulation of WNT2 transcripts in ESCC tumor samples, exclusive of any other known WNT2 related transcripts. mRNA abundance for known WNT pathway genes were collected from the RNAseq samples and were subjected to unsupervised hierarchical clustering. (b) Similar analyses were carried out for MAPK and AKT pathway genes identified by published literature curation.

a

	Normal	Tumor
-	4	1
+	5	2
++	1	5
+++	0	2
PR	60%	90%

PR: positive rate
Chi-square test: p < 0.05

b

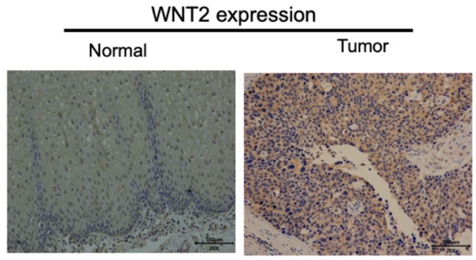

c

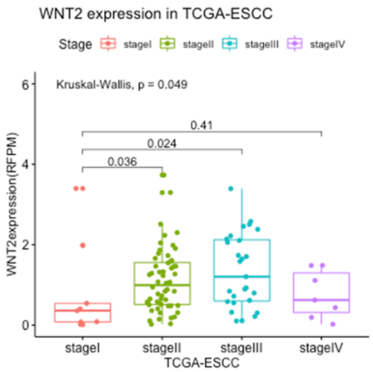

Supplementary Figure 22. WNT2 expression in tumors and its association with tumor stage. The immunohistochemistry assay was performed on histopathological slides from 10 independent ESCC patient's tumor and adjacent normal tissues. (a) The intensity of WNT2 protein expression was quantified as four categories (0: no expression, +: week expression, ++: medium expression, +++: strong expression). The positive rate of WNT2 expression was calculated as all positive cases/total cases. Chi-square test, $p < 0.05$. (b) Representative immunohistochemistry slides show strong intensity of WNT2 staining (predominantly in cytoplasm) in ESCC tumors than in matched normal tissue. 20x magnification, scale bar: 100um. (c) The association of WNT2 expression and tumor stages. TCGA-ESCC RNAseq was examined for WNT2 expression and its associate with clinical stages [I (n=10), II (n=54), III (n=17) and IV (n=7)]. Box and whisker plot: center line, median; box limits, upper and lower quartiles; and whiskers, maximum and minimum values. Kruskal-Wallis one-way analysis of variance method was used for multi-group non-parametric mean comparison.

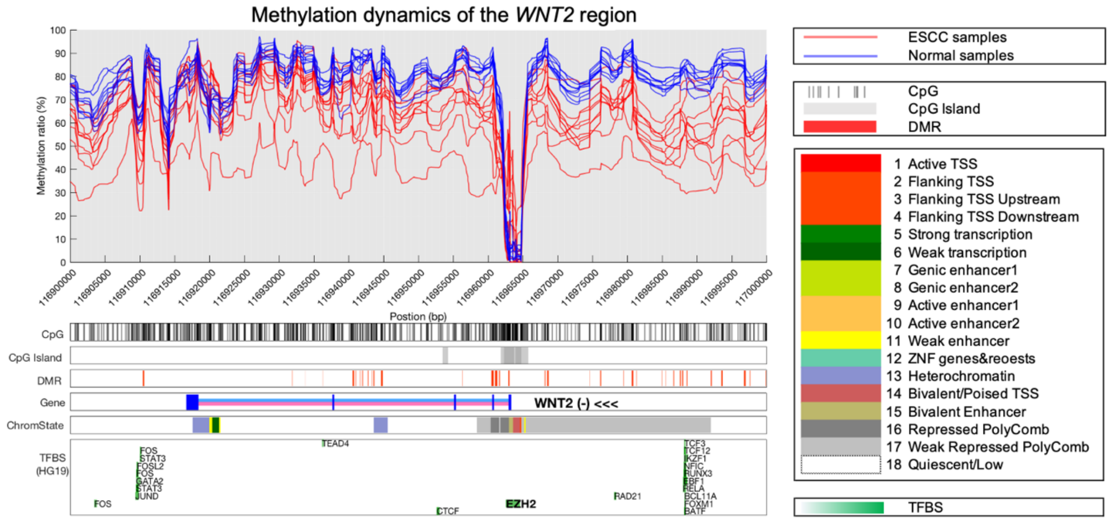

Supplementary Figure 23. Methylation around WNT2 gene promoter and gene body region. The DNA methylation status within the genomic regions (Chr7:116,900,000 – 117,000,000) in normal (blue lines) and ESCC tumors (red lines) (top panel) is shown. Six tracks showed individual CpGs, CpG island, DMRs, gene(s), chromatin states, and putative transcription factor binding within the selected regions. Detailed annotations for chromatin states are described on the right panel.

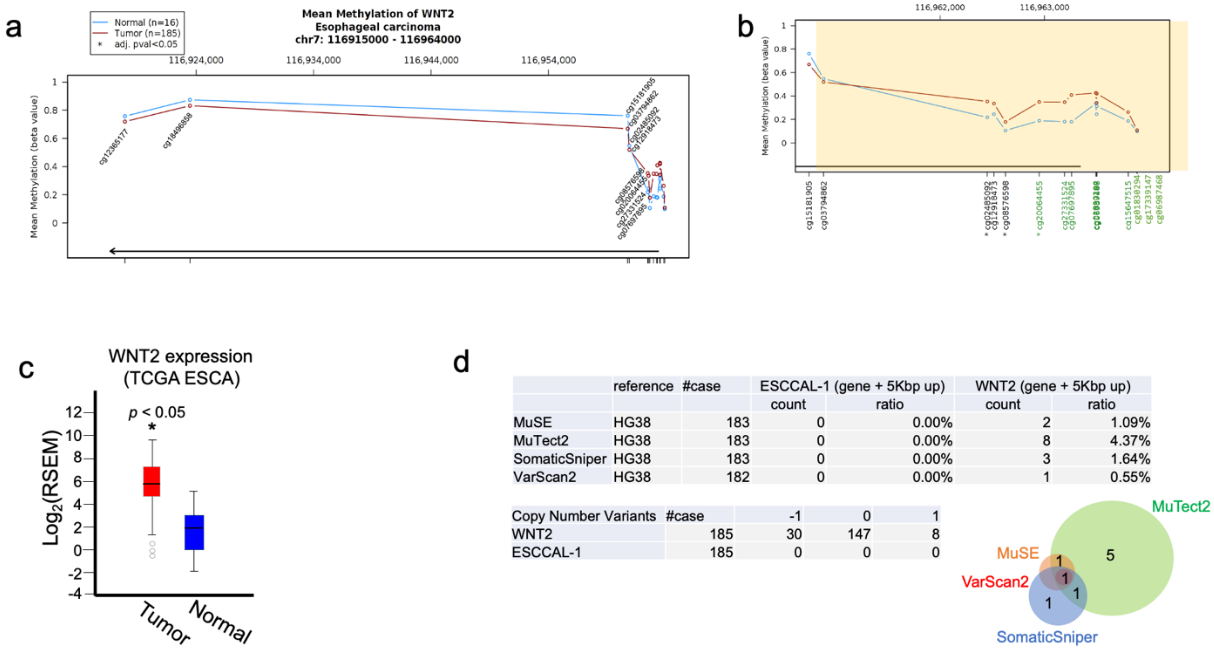

Supplementary Figure 24. Hypermethylation of the WNT2 promoter region and WNT2 high gene expression in TCGA-ESCA dataset. (a) The WNT2 promoter methylation alterations in TCGA-ESCA measured by HM450K array is shown. (b) Magnified region from (a) is shown. Green probes are CpG islands, p-values of * probes are less than 0.05. (c) WNT2 gene expression is increased in tumor samples (n= 186, red box) in TCGA-ESCA compared to normal samples (n=16, blue box), Statistical significance was assessed by unpaired two-sided t-test, * p-value < 0.05. Box and whisker plot: center line, median; box limits, upper and lower quartiles; and whiskers, maximum and minimum values. (d) List of the mutation and copy number alterations for WNT2 and ESCCAL-1 from TCGA ESCA DNA sequence dataset.

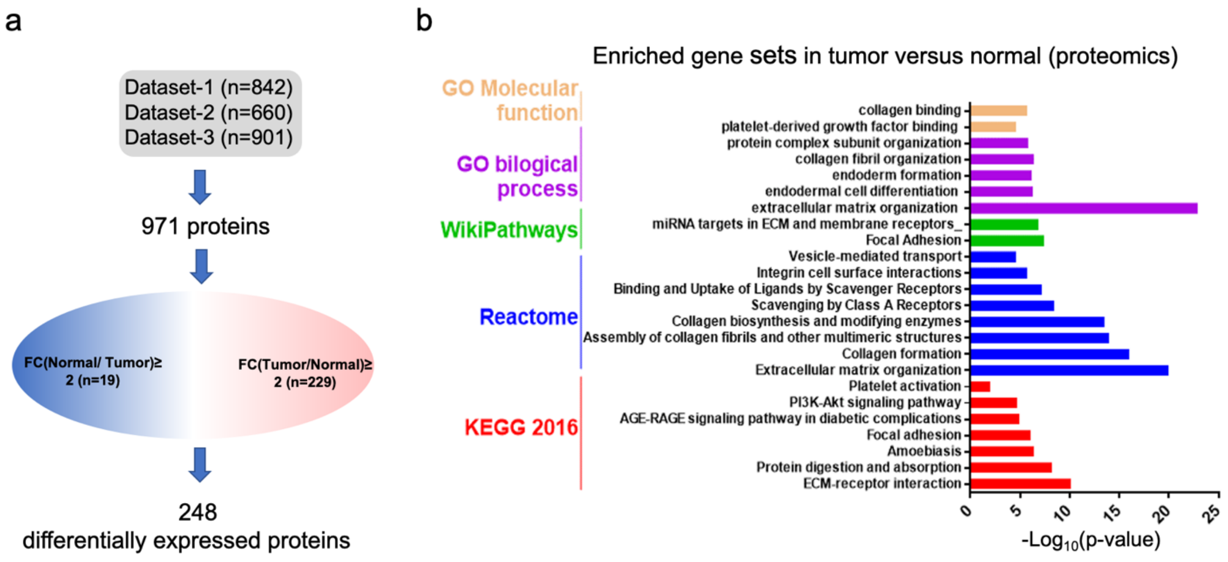

Supplementary Figure 25. Functional annotation of the protein expression from proteomic data.

(a) Ten paired normal and ESCC tumor samples were run in quantitative proteomics platform iTRAQ (Isobaric tag for relative and absolute quantitation) resulting in 3 datasets (Supplementary Data file 6). Datasets were merged into a combined union dataset from which 248 differentially expressed proteins were determined. (b) Functional annotation of these differentially expressed proteins was carried out in multiple databases using hypergeometric test (e.g. GO molecular function, GO biological process, WikiPathways, Reactome, KEGG 2016, BIOGRID etc.) The data are represented as a bar diagram indicating probability of the gene set enrichment. $-Log_{10}$(p-value) showing the significance of enrichment on the x-axis. Adjusted p value cutoff for significant gene sets was set to 0.01 ($-Log_{10}$ (p-value) >2). Hypergeometric test was used for pathway enrichment analysis within the algorithm.

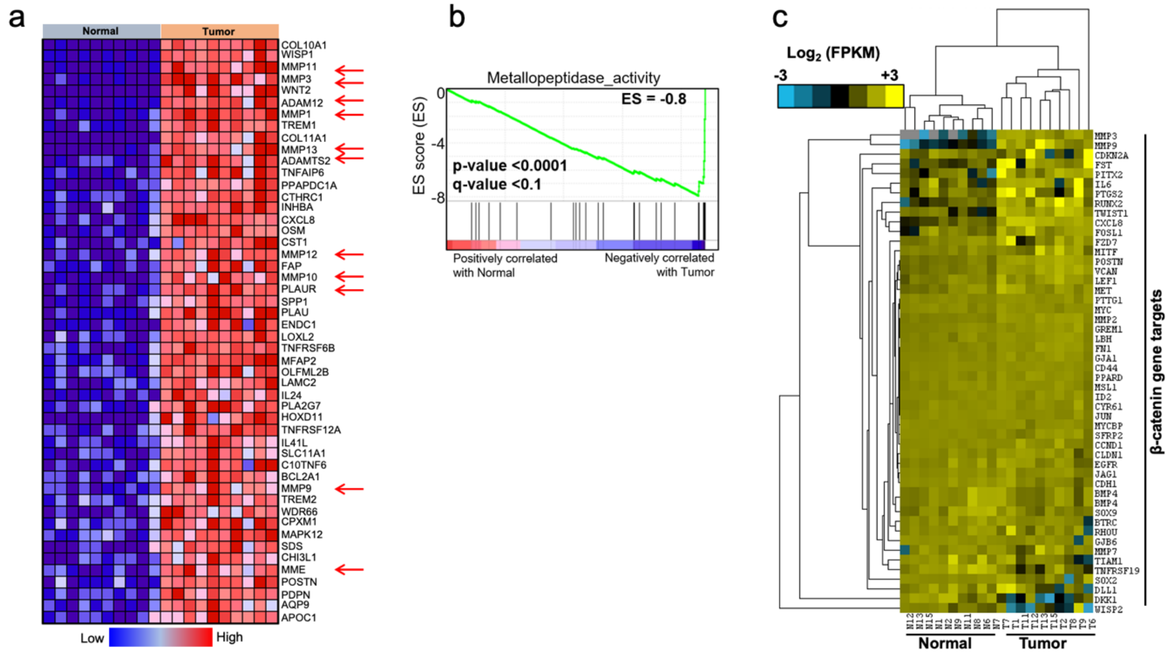

Supplementary Figure 26. A matrix metalloproteinase (MMP) program is upregulated in ESCC tumors. (a) Gene set enrichment analysis (GSEA) using the Broad Institute GSEA bioinformatic tool (Subramanian A. *et al.* PNAS, 2005. PMID:16199517) was carried out on RNAseq data from the tumor and normal samples. The data indicate enrichment of several MMPs and cell adhesion related gene transcripts in the leading edge of the tumor samples (b) Enrichment of 'Metalloprotease activity' in tumor samples is shown. Permutation test was used to estimate the enrichment score of gene set by GSEA algorithm, p-value <0.0001, q-value < 0.1. (c) Unsupervised hierarchical clustering analysis for mRNA abundances of known β-catenin target genes from RNAseq dataset.

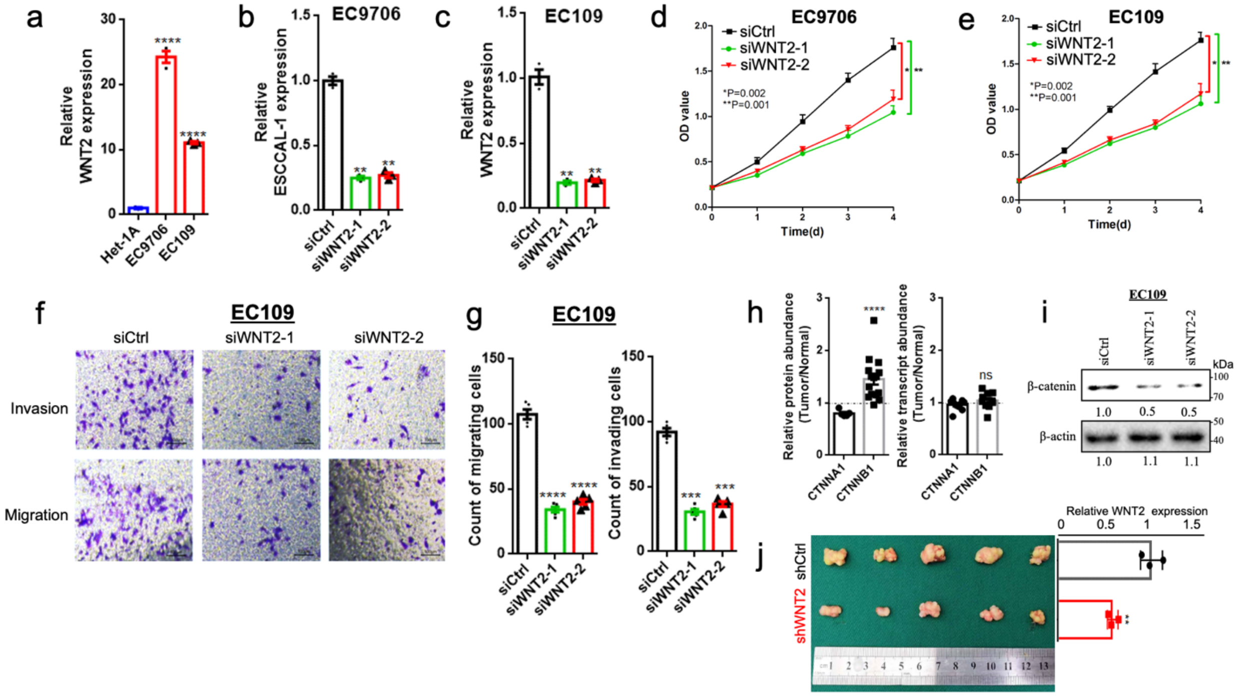

Supplementary Figure 27. Functional studies of WNT2 in ESCC. (a) Higher expression of WNT2 in ESCC cancer cell lines (EC9706 and EC109) relative to a normal esophageal epithelial cell line (Het-1A). **** p-value < 0.0001. (b) The efficiency of siRNA mediated knock-down of WNT2 in the EC9706 ESCC tumor cell line. ** p-value < 0.01. (c) The efficiency of WNT2 knock-down in EC109 ESCC tumor cell line, ** p-value < 0.01. (d) The inhibition of cellular proliferation in EC9706 cells with WNT2 knock-down (color lines) is shown. ** p-value < 0.01. (e) The inhibition of cellular proliferation in EC109 cells with WNT2 knock-down (color lines) is shown. ** p-value < 0.01. (f) Trans-well migration and invasion assay using EC109 cells in the presence or absence of WNT2 knock-down. Scale bar:100μm. Representative results from independent experiments (n=3). (g) Quantification of invasion and migration data from f with different microscopic fields (n=5 for each condition). *** p-value < 0.005, **** p-value < 0.001. (h) Total mRNA and protein expression of CTNNA1 and CTNNB1 in ESCC tumors relative to normal tissues measured by proteomic array (left panel) and RNAseq analyses (right panel) are shown. **** p-value < 0.001. ns: no significance. (i) Western blot showing decreased β-catenin protein expression in the ESCC tumor cell line EC109 using two independent siRNAs against WNT2. Data represent three independent experiments. (j) WNT2 knockdown xenograft mouse tumor formation. EC9706 cells expressing either shCtrl or shWNT2 were inoculated (s.c) into immunocompromised mice, the xenograft tumors were removed after 22 days of experiment. The right panel show the detection of WNT2 expression in removed tumor samples. ** p-value < 0.01. n=3 in a-e, j. Bar plots indicate mean ± sd, statistical significance was assessed by unpaired two-sided student's t-test.

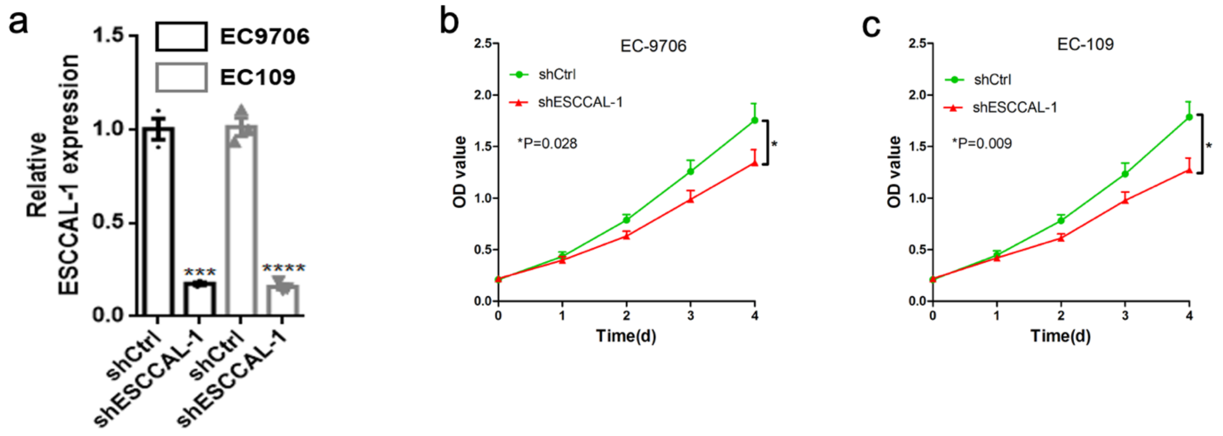

Supplementary Figure 28. ***In vitro*** **functional study of the lncRNA ESCCAL-1.** (a) The efficiency of knockdown ESCCAL-1 using shRNA in two different ESCC tumor cell lines (EC9706 and EC109. (b, c) The level of proliferation inhibition upon ESCCAL-1 knock-down in ESCC cell lines EC9706 and EC109 is shown. n=3 in a-c, bar plots indicate mean ± sd, statistical significance was assessed by unpaired two-sided student's t-test, * p-value < 0.05, *** p-value < 0.005, **** p-value < 0.0001.

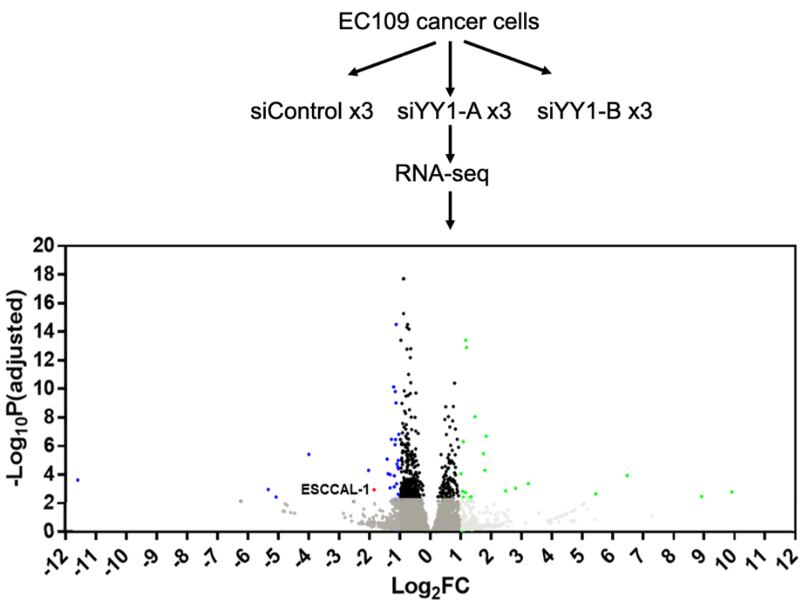

Supplementary Figure 29. Causative regulation of YY1-mediated ESCCAL-1 expression in ESCC.

EC109 cancer cells were transfected with either siRNA control or siYY1 for 72 hours, total RNA was harvested from triplicates of each conditions for RNA-seq assay. The differentially expressed genes (DEGs) from cells expressing shYY1 versus siControl were shown in a Volcano plot. The criteria for DEGs is to meet both $|Log_2FC| > 1$and adjusted p-value < 0.05. Each dot represents a gene, blue dots are significantly downregulated genes, green dots are significantly upregulated genes in cells with YY1 knockdown. ESCCAL-1 is highlighted as red dot as one of the most significantly down-regulated genes.

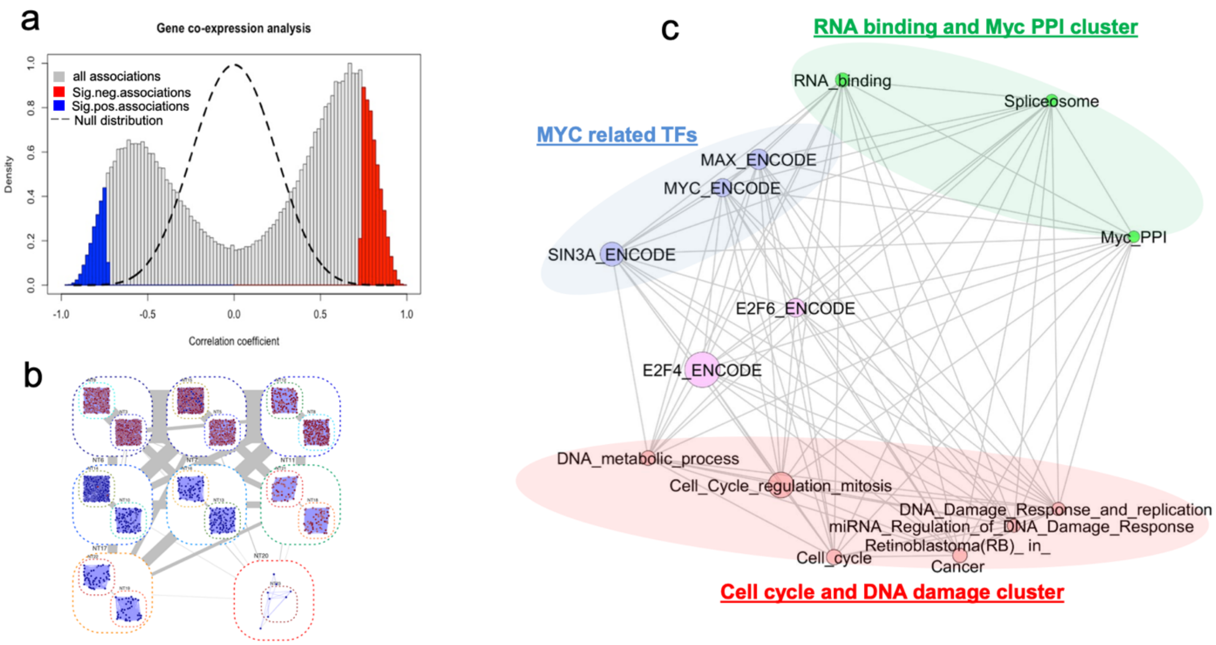

Supplementary Figure 30. Co-expression of lncRNA ESCCAL-1 with coding genes derived from the RNA-seq dataset. (a) The correlation distribution with significant associations of gene expression is shown. 3554 differentially expressed genes between ESCC and normal tissues measured by RNAseq (|Log_2(fold change) >=1 and FDR<0.05) were used as input for co-expression analysis using RedeR algorithm (Castro M.,*et al*. Genome Biology. 2012. PMID: 22531049). Red color represents significant positive correlation, blue color represents significant negative correlation. 1000 iteration was computed, significant association of p-value < 0.001. (b) The resulting clusters were nested to clear the visualization. The nests are numbered from NT 1 to NT 23. The width of gray lines between nodes represents the number of interactions among clusters. Red nodes represent increased transcription in ESCC tumors compared to normal tissues and blue nodes represent decreased transcription in ESCC tumors compared to normal tissues. The dashed lines enclose clusters of co-expressed genes. (c) The significantly co-expressed genes associated with lncRNA ESCCAL-1 expression were used for the functional annotation analysis using multiple curated databases (BIOGRID, ENCODE, KEGG, Reactome, GO molecular function). The gene sets are represented as nodes and node size represents the significance of the hypergeometric analysis overlapping of ESCCAL-1 co-expressed genes with curated gene sets. Genes in specific pathways with higher significance for the overlap is indicated by node proximity. 2D projection of 3D network clustering using edge weights were performed using edge-directed spring embedded network layout in Cytoscape 3.2. Ellipses were drawn manually for visualization to demarcate the clusters and nodes were further color coded manually based on their molecular properties. Overexpression of ESCCAL-1 correlated with dysregulation of the cell cycle, DNA repair, RNA binding processing and Myc pathway activation.

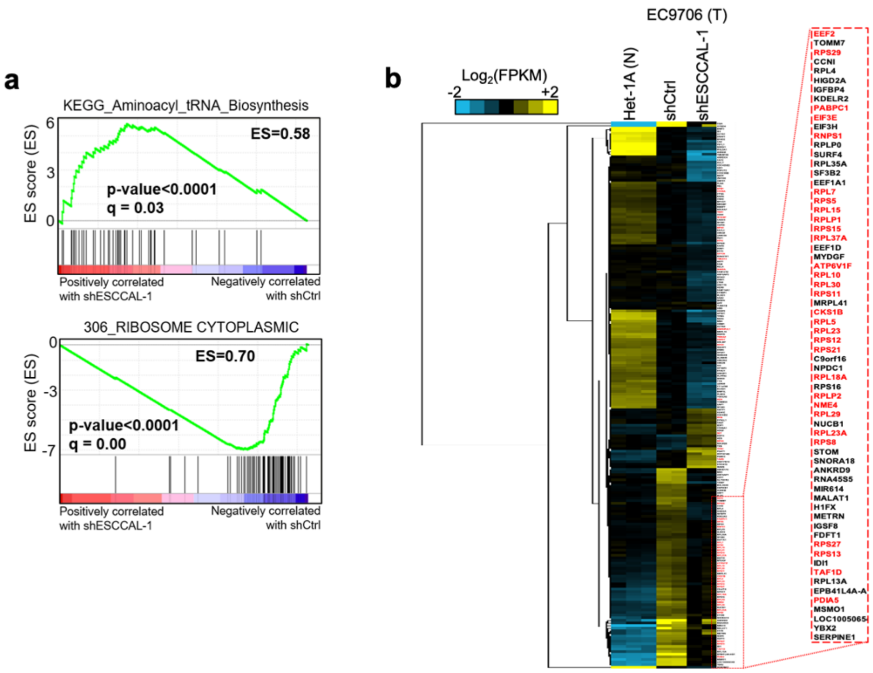

Supplementary Figure 31. Mechanistic understanding of ESCCAL-1-mediated downstream biology. (a) Gene set enrichment analysis (GSEA) using the RNAseq dataset derived from an immortalized normal esophageal epithelial cell line (Het-1A) and an ESCC tumor cell line EC9706 expressing shRNA control or EC9706 cells expressing shRNA targeting ESCCAL-1. Two replicates of each cohort of transcripts were subjected to GSEA platform (Broad Institute) using curated gene sets (Subramanian A. *et al.* PNAS, 2005. PMID:16199517). The analysis indicated upregulation of "KEGG AMINOACYL_TRNA_BIOSYNTHESIS" and downregulation of "RIBOSOME_CYTOPLASMIC" gene sets on ESCCAL-1 silenced cohort. Permutation test was used to estimate the enrichment score of gene set by GSEA algorithm, p-value < 0.0001, q < 0.05. (b) Unsupervised hierarchical clustering of significant differentially expressed gene clusters in Het-1A (normal) versus shControl expressed EC9706 versus shESCCAL-1 expressed EC9706. Differentially expressed genes were selected based on an iterative clustering approach selecting for genes showing the top 5% of significant variable and differential gene expressions. 210 selected genes were subjected to a hierarchical clustering using the Euclidean distance metric and represented as a heatmap where blue represents lower expression and yellow represents higher expression. The fonts of gene names were further color coded. For example, red represents enrichment of Myc gene sets derived from the ENCODE project (see main Figure 7i).

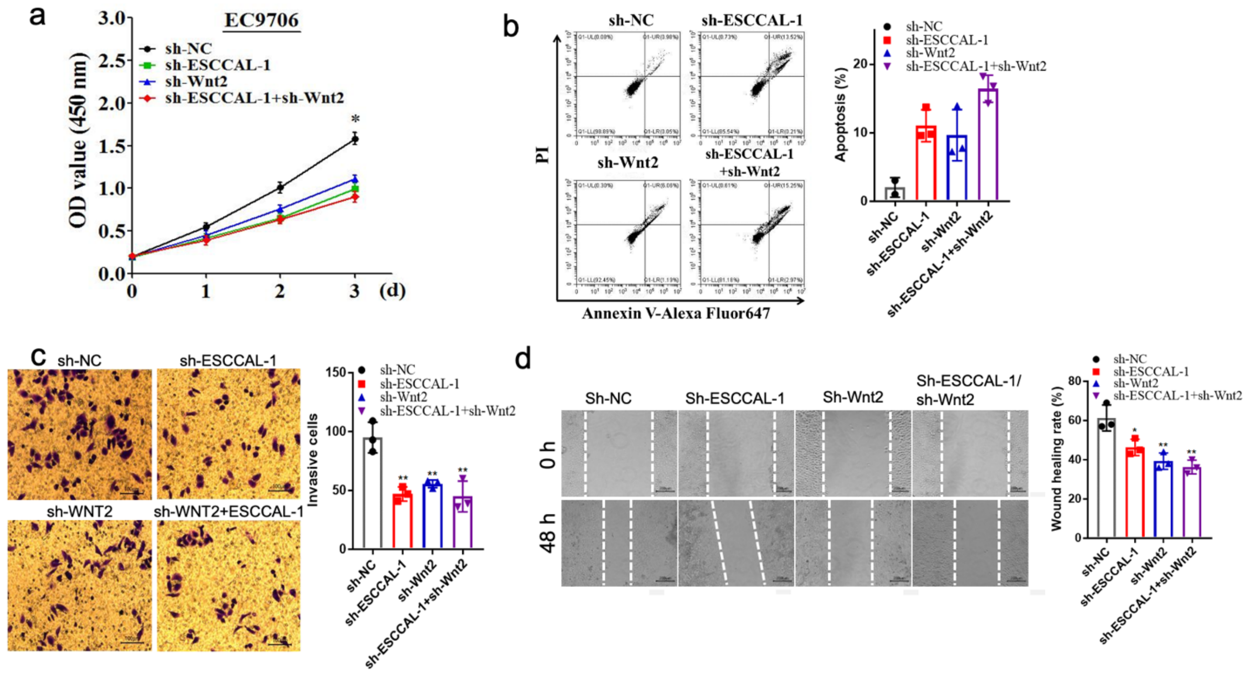

Supplementary Figure 32. ***In vitro*** **functional study of simultaneous knockdown of WNT2 and ESCCAL-1 in ESCC cancer cells.** EC9706 cancer cells were transfected with either shESCCAL-1, siWNT2 or both for 48 hours as well as corresponding controls. The effects of proliferation (a), apoptosis (b), invasion (c) and wound healing (d) assay were performed in each single knockdown or double knockdown conditions. The quantification in b, c, d from three independent experiments is shown beside representative images. N=3 in a-d, bar plots indicate mean ± sd, statistical significance was assessed by unpaired two-sided student's t-test, * p-value < 0.05, *** p-value < 0.005, **** p-value < 0.0001. Scale bar: 100μm in c, 200μm in d.

Supplementary Table 1. All primers were used in the study

GENE	Primer name	Primer sequence
ESCCAL-1	q-PCR primer	Forward:5'-CCAGACAGCAGCAAAGCAAT-3'Reverse:5'-GGAAGCAGCAAATGTGTCCAT-3'
GAPDH	q-PCR primer	Forward:5'-TCAGAGGACGGCATGAGACTTA-3'Reverse:5'-AGCAGGACCCAGGTGTCATT-3'
ESCCAL-1	methylated primer	Forword:5'-TGCGCCAGCCGAAGCAGGGCGA-3' Reverse:5'-CGAGACTCCGTGGGCGTA-3'
	unmethylated primer	Forword:5'-TGTGTTAGTTGAAGTAGGGTGA-3'Reverse:5'-CAAAACTCCATAAACATA-3'
WNT2	q-PCR primer	Forward:5'-GAATGGCTTTCAATACCCTT-3'Reverse:5'-CTGAGGCTTTTGCTCTTACA-3'
WNT2	methylated primer	Forward: 5'-CGAGACCATGGTGAAACCCCG-3' Reverse: 5'-CGTGATATGGGCTCACTGCAA-3'
	unmethylated primer	Forward: 5'-TGAGATTATGGTGAAATTTTG-3' Reverse: 5'-CATAATATAAACTCACTACAA-3'
WNT2	Si-WNT2-1	GCGCAUUUGUGGAUGCAAATT UUUGCAUCCACAAAUGCGCTT
	Si-WNT2-2	CAUUGACUAUGGGAUCAAATT UUUGAUCCCAUAGUCAAUGTT
	Si-NC	UUCUCCGAACGUGUCACGUTT ACGUGACACGUUCGGAGAATT
ESCCAL-1-YY1	CHIP primer	Forward:5'-TTTGAAATAATGAGTTATGAG-3'Reverse:5'-GGAAGCAGCAAATGTGTCCAT-3'
WNT2-EZH2	CHIP primer	Forward:5'-AGCTGTGCGTCTTTTGACCT-3' Reverse:5'-GGAAGAAAGCTAGAGCTGGAAC-3'
YY1	si-NC	UUCUCCGAACGUGUCACGUTT ACGUGACACGUUCGGAGAATT
	si-YY1-1	ACAGAAAGGGCAACAAUAATT UUAUUGUUGCCCUUUCUGUTT
	si-YY1-2	AAGAUGAUGCUCCAAGAACTT GUUCUUGGAGCAUCAUCUUTT
	si-YY1-3	GAACUCACCUCCUGAUUAUTT AUAAUCAGGAGGUGAGUUCTT
