# Supplementary material for: Multi-faceted epigenetic dysregulation of gene expression promotes esophageal squamous cell carcinoma
Source: Nat Commun. 2020 Jul 22;11:3675. doi: 10.1038/s41467-020-17227-z (PMC7376194; doi:10.1038/s41467-020-17227-z)
Supplement: Supplementary file 3 — Description of Additional Supplementary Files [file 41467_2020_17227_MOESM3_ESM.pdf]

Description of Additional Supplementary Files

File Name: Supplementary Data 1
Description: The processed whole genome bisulfite sequencing data.

File Name: Supplementary Data 2
Description: Transcription factor binding consensus distributions in the genome from the ENCODE project.

File Name: Supplementary Data 3
Description: Enrichment datasets from genomic region enrichment analysis for hyper-DMRs and hypo-DMRs.

File Name: Supplementary Data 4
Description: C1-C4 clusters of promoter methylation changes and corresponding gene expression.

File Name: Supplementary Data 5
Description: The most significant 210 of the differentially expressed genes from shESCCAL-1 knockdown studies and RNA-seq analysis.

File Name: Supplementary Data 6
Description: The differentially expressed proteins arising from the normal-ESCC tumor sample proteomic data analysis.
