# Supplementary material for: Multi-faceted epigenetic dysregulation of gene expression promotes esophageal squamous cell carcinoma
Source: Nat Commun. 2020 Jul 22;11:3675. doi: 10.1038/s41467-020-17227-z (PMC7376194; doi:10.1038/s41467-020-17227-z)
Supplement: Supplementary file 10 — Reporting Summary [file 41467_2020_17227_MOESM10_ESM.pdf]

nature research

Corresponding author(s): Wei Cao
Wei Wu
Michael Snyder
Trever G. Bivona (leading)

Last updated by author(s): Jun 4, 2020

Reporting Summary

Nature Research wishes to improve the reproducibility of the work that we publish. This form provides structure for consistency and transparency in reporting. For further information on Nature Research policies, see Authors & Referees and the Editorial Policy Checklist.

Statistics

For all statistical analyses, confirm that the following items are present in the figure legend, table legend, main text, or Methods section.

n/a	Confirmed	
☐	☒	The exact sample size (*n*) for each experimental group/condition, given as a discrete number and unit of measurement
☐	☒	A statement on whether measurements were taken from distinct samples or whether the same sample was measured repeatedly
☐	☒	The statistical test(s) used AND whether they are one- or two-sided<br>*Only common tests should be described solely by name; describe more complex techniques in the Methods section.*
☐	☒	A description of all covariates tested
☐	☒	A description of any assumptions or corrections, such as tests of normality and adjustment for multiple comparisons
☐	☒	A full description of the statistical parameters including central tendency (e.g. means) or other basic estimates (e.g. regression coefficient) AND variation (e.g. standard deviation) or associated estimates of uncertainty (e.g. confidence intervals)
☐	☒	For null hypothesis testing, the test statistic (e.g. *F*, *t*, *r*) with confidence intervals, effect sizes, degrees of freedom and *P* value noted<br>*Give P values as exact values whenever suitable.*
☒	☐	For Bayesian analysis, information on the choice of priors and Markov chain Monte Carlo settings
☒	☐	For hierarchical and complex designs, identification of the appropriate level for tests and full reporting of outcomes
☐	☒	Estimates of effect sizes (e.g. Cohen's *d*, Pearson's *r*), indicating how they were calculated

Our web collection on statistics for biologists contains articles on many of the points above.

Software and code

Policy information about availability of computer code

Data collection

Whole Genome Sequencing (WGS): Base calling was performed with the Illumina Real Time Analysis version 2.7.7 and the output was demultiplexed and converted to FastQ format with the Illumina Bcl2fastq v2.19.0.316. The FastQC package (http://www.bioinformatics.babraham.ac.uk/projects/fastqc) was used to check the quality of the sequencing reads. Sequencing adapters were trimmed from raw reads with Trimmomatic (version 0.36). Mapping, marking duplicates marking, and mutation calling were carried out with bcbio-
nextgen (https://github.com/bcbio/bcbio-nextgen). Specifically, Reads were mapped to the human reference genome assembly GRCh37 using BWA-MEM in the BWA package (version 0.7.17) with the default parameters. Duplicates were marked using the tool Sambamba version 0.6.6.
Whole Genome Bisulfite sequencing (WGBS) The reads were trimmed using Trim Galore! (v0.4.1) BSMAP (v2.89) was used to align the trimmed reads to HG19 reference genome. SAMtools (v. 1.3.1) was used to sort by genomic coordination and make a bam file index. Picard Tools (v.1.92) was used to remove PCR duplicates. MOABS (v. 1.3.4) was used compute the methylation ratio per CpG with the option of '--cytosineMinScore 20 --skipRandomChrom 1 -p 4 --keepTemp 0 --processPEOverlapSeq 1 --requiredFlag 2 --excludedFlag 256 --minFragSize 110 --reportCpX G --qualityScoreBase 0 --trimRRBSEndRepairSeq 0 --trimWGBSEndRepairPE1Seq 5 --trimWGBSEndRepairPE2Seq 5'.
Whole transcriptome sequencing (RNA-seq) RNA-Seq reads were mapped to the HG19 reference genome using STAR (Spliced Transcripts Align to a Reference, v2.4.2a). The expression level of transcript per million (TPM) reads were quantified using RNA-Seq by Expectation-Maximization algorithm (RSEM v1.2.29).
Chromatin Immunoprecipitation (ChIP) with massively parallel DNA sequencing (ChIP-seq)
Quality control of the sequencing data was performed using Sickle (https://github.com/najoshi/sickle) and SeqPrep (https://github.com/jstjohn/SeqPrep). The sequencing output raw reads were trimmed by stripping the adaptor sequences and ambiguous nucleotides and reads with quality scores less than 20 and lengths below 20bp were removed.
Proteomic assay and data analysis (Isobaric tag for relative and absolute quantitation, iTRAQ)
The isobaric labeling and LC-MS quantifications were operated in Beijing Genomics Institute (BGI) using optimized quantitative MS-MS

protocols. IQuant was used to identify protein and for data analysis (http://sourceforge.net/projects/iquant/).

Data analysis

Whole Genome Sequencing (WGS): Somatic mutations include single nucleotide variants (SNVs), small insertions and/or deletions (indels), and structural variants (SVs). The detection of somatic mutations was performed using tumor and matched normal whole genome BAM files generated in the steps described above. We used a series of software packages including VarDict (https://github.com/AstraZeneca-NGS/VarDict
), MuTect2 (version 4.0) and Strelka2 (version 2.9) to detect somatic SNVs and indels, and packages including LUMPY (version 0.2.13), Manta (https://github.com/Illumina/manta
), CNVkit (version 0.9.5) and MetaSV to detect SVs.
Whole Genome Bisulfite sequencing (WGBS) Entropy is computed per CpG in both ESCC and normal esophageal cohorts separately as a measure of variance. The 'entropy' function was used in the 'stats' package of SciPy (v 0.19.1) on top of python3 (v 3.5.2). The bin size was 10%. The distribution of CpG entropy was plotted using MatLab (v. 9.2) 'plot' and 'histogram' function with the default option.
Differentially methylated regions (DMRs) was computed from ~5Ms of confident differentially methylated CpGs. The window size is flexible as long as any two CpGs locate in 150bp and have consistent methylation pattern either keeping hypermethylated or hypomethylated. The criteria make sure the minimum CpG density is at least 0.01. DMRs peak size is of 150-350bp and CpG density peak is of 0.04-0.05 (Supplementary Figure 7a, b). The genomic region enrichment analysis for DMRs was conducted using LOLA package (http://code.databio.org/LOLA) in R.

Whole transcriptome sequencing (RNA-seq) The quantified gene expressions of 26,334 transcripts (including coding genes and non-coding genes) were processed in Rstudio console with R programme (v 3.4). Differentially expressed genes between tumor and normal samples were identified using the EdgeR algorithm.
Chromatin Immunoprecipitation (ChIP) with massively parallel DNA sequencing (ChIP-seq)
The cleaned reads were aligned to human reference genome hg19 using BWA. MACS2 (model-based analysis of ChIP-seq) algorithm was used for peak calling. The reads of EZH2 binding on WNT2 promoter region were visualized using Integrative Genomics Viewer (IGV, Broad Institute).
Proteomic assay and data analysis (Isobaric tag for relative and absolute quantitation, iTRAQ)
For improved protein identification, a Mascot Percolator and Mascot Parser (https://www.sanger.ac.uk/science/tools/mascotpercolator), a customized post-processing tool was used. The signal to noise ratio was decreased by variance stabilization normalization (VSN).
Co-expression analysis of RNAseq Co-expression analysis was conducted in R environment using RedeR package.
Copy number alteration inferred from RNAseq CNVkit-RNA (https://www.biorxiv.org/content/10.1101/408534v1) was used to infer copy number alterations from RNAseq reads. The segments and recurrent copy number gains or loss across samples were generated and plotted using GISTIC 2.0 algorithm.

For manuscripts utilizing custom algorithms or software that are central to the research but not yet described in published literature, software must be made available to editors/reviewers. We strongly encourage code deposition in a community repository (e.g. GitHub). See the Nature Research guidelines for submitting code & software for further information.

Data

Policy information about availability of data

All manuscripts must include a data availability statement. This statement should provide the following information, where applicable:

- Accession codes, unique identifiers, or web links for publicly available datasets
- A list of figures that have associated raw data
- A description of any restrictions on data availability

All data generated or analysed during this study are included in this published article (and its supplementary information files). The WGBS, RNAseq and ChIPseq raw data is submitting to GEO, and WGS is submitting to NCBI sequencing read archive (SRA) database. Accession code GSE149612 is for WGBS, RNAseq and ChIPseq; accession code PRJNA630082 is for WGS.

Field-specific reporting

Please select the one below that is the best fit for your research. If you are not sure, read the appropriate sections before making your selection.

☒ Life sciences ☐ Behavioural & social sciences ☐ Ecological, evolutionary & environmental sciences

For a reference copy of the document with all sections, see nature.com/documents/nr-reporting-summary-flat.pdf

Life sciences study design

All studies must disclose on these points even when the disclosure is negative.

Sample size

Based on study of epigenome-wide association scans with methylation array (Pei-Chien Tsai and Jordana T bell. International Journal of Epidemiology. 2015; 44(4): 1429-1441), estimated 10 samples are required to reach 80% power at nominal significance of $p = 0.05$ with effect size of mean differences of 15% in case and control designs. For cancer methylome study, the effect size between normal and tumor is at lease 20%, therefore we designed to collect 10 paired esophageal squamous cell carcinoma and adjacent normal samples for whole genome bisulfite sequencing study and matching RNAseq.

Data exclusions

One whole genome bisulfite sequencing data from normal sample was excluded from downstream analysis because the sequencing coverage did not meat our cut-off of 15X.

Replication

For tissue sequencing data,ten patients represent different biological replicates and we verified our findings on TCGA-esophageal cancer

Replication	methylation array data with 450K CpG probes and independent tissue samples and cell lines. Other experimental data, we performed duplicate or triplicate technical or biological repeats.
Randomization	This is not relevant to this study.
Blinding	This is not relevant to this study

Reporting for specific materials, systems and methods

We require information from authors about some types of materials, experimental systems and methods used in many studies. Here, indicate whether each material, system or method listed is relevant to your study. If you are not sure if a list item applies to your research, read the appropriate section before selecting a response.

Materials & experimental systems

n/a	Involved in the study	
☐	☒	Antibodies
☐	☒	Eukaryotic cell lines
☒	☐	Palaeontology
☐	☒	Animals and other organisms
☒	☐	Human research participants
☒	☐	Clinical data

Methods

n/a	Involved in the study	
☐	☒	ChIP-seq
☐	☒	Flow cytometry
☒	☐	MRI-based neuroimaging

Antibodies

Antibodies used	anti-KMT6/EZH2 antibody (ab195409, Abcam), Anti-Histone H3 (acetyl K27) antibody ChIP Grade (ab4729,Abcam), Anti-YY1 antibody (ab38422,Abcam), rabbit anti-histone H3 (a technical positive control; 1:50) (catalog no. 4620; Cell Signaling Technologies), and normal rabbit IgG (a negative control; 5 μg) (catalog no. 2729; Cell Signaling Technology, USA), antibodies WNT2 (Bioworld, USA). anti-MMP3 (Rabbit antibody, catalog no. 14351; Cell Signaling Technology, USA), anti-MMP9 (Rabbit antibody, catalog no. 13667, Cell Signaling Technology, USA).
Validation	There antibodies are purchased from companies, these antibodies are validated by manufactures.

Eukaryotic cell lines

Policy information about cell lines

Cell line source(s)	Human ESCC cell lines (EC-109, EC-9706, EC-1) and immortalized esophageal epithelial cell line Het-1A were purchased from the Shanghai Institutes for Biological Science (Shanghai, China).
Authentication	There are commercially available cell lines.
Mycoplasma contamination	These cell lines had no mycoplasma contamination during experiments in this study.
Commonly misidentified lines (See ICLAC register)	None

Animals and other organisms

Policy information about studies involving animals; ARRIVE guidelines recommended for reporting animal research

Laboratory animals	Six-week old male BALB/c immunodeficient mice were purchased from the Shanghai Experimental Animal Center, Chinese Academy of Sciences (Shanghai, China).
Wild animals	NA
Field-collected samples	NA
Ethics oversight	Animal experimental procedures were carried out according to the Ethical Committee of Zhengzhou University.

Note that full information on the approval of the study protocol must also be provided in the manuscript.

ChIP-seq

Data deposition

☒ Confirm that both raw and final processed data have been deposited in a public database such as GEO.

☒ Confirm that you have deposited or provided access to graph files (e.g. BED files) for the called peaks.

Data access links<br>*May remain private before publication.*	*For "Initial submission" or "Revised version" documents, provide reviewer access links. For your "Final submission" document, provide a link to the deposited data.*
Files in database submission	Sequencing fastq files and called peaks in bed files
Genome browser session<br>(e.g. UCSC)	*Provide a link to an anonymized genome browser session for "Initial submission" and "Revised version" documents only, to enable peer review. Write "no longer applicable" for "Final submission" documents.*

Methodology

Replicates	Immortalized esophagus epithelial cell line Het-1A and ESCC cell line EC109 was used for ChIPseq, n=1 for each condition.
Sequencing depth	Both EZH2 and IgG pull-down DNAs were sequenced with the length in 151 bp, paired-end. Reads in EC109-EZH2, Het-1A-EZH2 are 18.4 millions and 19.6 millions, respectively. 52.5% are properly mapped reads.
Antibodies	anti-KMT6/EZH2 antibody (ab195409, Abcam), and normal rabbit IgG (a negative control; 5 μg) (catalog no. 2729; Cell Signaling Technologies).
Peak calling parameters	As default setting
Data quality	EZH2 shows 398 and 996 peaks in EC109 cells and Het-1A, respectively, none of them are above 5 fold. We manually to examine the targeted regions using IGV and presented in the paper.
Software	BAW-MEM aligner was used for mapping, MACS2 algorithm was used for peak calling.

Flow Cytometry

Plots

Confirm that:

☒ The axis labels state the marker and fluorochrome used (e.g. CD4-FITC).

☒ The axis scales are clearly visible. Include numbers along axes only for bottom left plot of group (a 'group' is an analysis of identical markers).

☒ All plots are contour plots with outliers or pseudocolor plots.

☒ A numerical value for number of cells or percentage (with statistics) is provided.

Methodology

Sample preparation	Cell apoptosis was assessed using the AnnexinV/PI Kit (Abbkine. Ltd, Wuhan, China). The procedures were performed according to the user's instructions. Briefly, the EC9706 cells were seeded into 6-well plates at a concentration of 4×105 cells per well and incubated for 24h. After lentivirus transfection, cells were subjected to apoptosis detection using a flow cytometry (Cytoflex, Beckman, USA).
Instrument	Cytoflex, Beckman Counter, USA
Software	CytExpert
Cell population abundance	Esophageal Carcinoma Cells EC9706 cell line was used. There is not cell sorting in this experiment.
Gating strategy	FACS gating strategy for apoptotic cells, first plot gating for living cells, then second plot for apoptotic cells. The cells that were not treated with Annexin V-PI were used as controls.

☐ Tick this box to confirm that a figure exemplifying the gating strategy is provided in the Supplementary Information.
